# Glucocorticoid-dependent REDD1 expression reduces muscle metabolism to enable adaptation under energetic stress

**DOI:** 10.1186/s12915-018-0525-4

**Published:** 2018-06-12

**Authors:** Florian A. Britto, Fabienne Cortade, Yassine Belloum, Marine Blaquière, Yann S. Gallot, Aurélie Docquier, Allan F. Pagano, Elodie Jublanc, Nadia Bendridi, Christelle Koechlin-Ramonatxo, Béatrice Chabi, Marc Francaux, François Casas, Damien Freyssenet, Jennifer Rieusset, Sophie Giorgetti-Peraldi, Gilles Carnac, Vincent Ollendorff, François B. Favier

**Affiliations:** 1 DMEM, Univ. Montpellier, INRA, Montpellier, France; 2 PHYMEDEXP, Univ. Montpellier, INSERM, CNRS, CHRU of Montpellier, Montpellier, France; 3 LIBM, Univ. Lyon, Saint Etienne, France; 4 INSERM UMR-1060, CarMeN Laboratory, Lyon 1 University, INRA U1397, Oullins, France; 5 Institute of Neuroscience, Université catholique de Louvain, Louvain-la-Neuve, Belgium; 6 Université Cote d’Azur, INSERM, UMR1065, C3M, Nice, France

**Keywords:** Skeletal muscle, Metabolism, Hypoxia, Fasting, Exercise, Mitochondria, MAMs, Energy expenditure, mTOR

## Abstract

**Background:**

Skeletal muscle atrophy is a common feature of numerous chronic pathologies and is correlated with patient mortality. The REDD1 protein is currently recognized as a negative regulator of muscle mass through inhibition of the Akt/mTORC1 signaling pathway. REDD1 expression is notably induced following glucocorticoid secretion, which is a component of energy stress responses.

**Results:**

Unexpectedly, we show here that REDD1 instead limits muscle loss during energetic stresses such as hypoxia and fasting by reducing glycogen depletion and AMPK activation. Indeed, we demonstrate that REDD1 is required to decrease O_2_ and ATP consumption in skeletal muscle via reduction of the extent of mitochondrial-associated endoplasmic reticulum membranes (MAMs), a central hub connecting energy production by mitochondria and anabolic processes. In fact, REDD1 inhibits ATP-demanding processes such as glycogen storage and protein synthesis through disruption of the Akt/Hexokinase II and PRAS40/mTORC1 signaling pathways in MAMs. Our results uncover a new REDD1-dependent mechanism coupling mitochondrial respiration and anabolic processes during hypoxia, fasting, and exercise.

**Conclusions:**

Therefore, REDD1 is a crucial negative regulator of energy expenditure that is necessary for muscle adaptation during energetic stresses. This present study could shed new light on the role of REDD1 in several pathologies associated with energetic metabolism alteration, such as cancer, diabetes, and Parkinson’s disease.

**Electronic supplementary material:**

The online version of this article (10.1186/s12915-018-0525-4) contains supplementary material, which is available to authorized users.

## Background

Regulated in development and DNA damage (REDD1) is a small ubiquitous and conserved protein whose expression is low in basal conditions but highly increased in response to hypoxia, administration of glucocorticoids, endurance exercise, DNA damage, or the unfolding protein response [1–5]. REDD1 is involved in numerous pathologies including oxygen-induced retinopathy [6], emphysema [7], Parkinson’s disease [8], depression [9], diabetes [10], and cancer [11]. Many of these diseases are associated with an impairment of skeletal muscle function, i.e., decreased muscle mass and increased fatigability. Muscle function is directly correlated with patient quality of life and mortality [12, 13] and excessive muscle loss reduces the efficacy of pharmacological treatments [14].

Skeletal muscle function is partially controlled by the Akt/mammalian Target of Rapamycin Complex 1 (mTORC1) pathway. Akt/mTORC1 regulates both skeletal muscle mass and metabolism via activation of several ATP-demanding processes such as protein synthesis [15], glycogen storage [16, 17], and mitochondrial biogenesis [18]. The Akt/mTOR pathway also controls mitochondrial function by fostering the interaction between mitochondria and the endoplasmic reticulum (ER) in the mitochondria-associated membranes compartment (MAMs) [19]. Indeed, ER transfers Ca^2+^ to mitochondria [20], promoting O_2_ consumption through stimulation of several oxidative enzymes (reviewed in [21]). Conversely, mitochondria provide ATP to ER for macromolecule biosynthesis, notably proteins [22]. Therefore, activation of the Akt/mTOR pathway leads to an increased O_2_ uptake [23, 24] and ATP consumption [25]. mTORC1 activation is regulated by Akt-mediated inhibition of PRAS40 and TSC2 [26, 27]. Akt also promotes glycogen storage via mitochondrial association of Hexokinase II (HKII) and inhibition of Glycogen Synthase Kinase 3 (GSK3) [28, 29].

A recognized role for REDD1 is the inhibition of the Akt/mTORC1 pathway through TSC2 activation [30] or Akt inhibition [31]. Consistently, recent studies have demonstrated that REDD1 is required for mTORC1-dependent protein synthesis inhibition following fasting, sepsis, or dexamethasone (DEX) treatment [32–34]. In addition, REDD1 overexpression is responsible for skeletal muscle atrophy and mTORC1 inhibition [32, 35]. Interestingly, REDD1 expression increases during energetic stresses known to promote amyotrophy such as hypoxia and fasting [36, 37]. Moreover, a pool of REDD1 protein has been localized in the mitochondrial compartment in cultured cells [11], raising the question of its potential role in mitochondrial respiration or MAMs remodeling. Importantly, recent published data on O_2_ consumption in REDD1-knockout (KO) mouse embryonic fibroblasts (MEFs) gave contradictory results with either an increase [38] or a decrease [39] in basal respiration. However, although recent reviews suggest a metabolic role for REDD1 [40, 41], its physiological role in O_2_ consumption and adaptation to energetic stress (hypoxia, fasting, and exercise) remains to be elucidated.

We report here that REDD1 expression spares skeletal muscle mass during hypoxia and fasting. We propose that one physiological role of REDD1 is to decrease energetic demand by reducing the mitochondria/ER interaction and by downregulating ATP-dependent synthesis pathways to enable skeletal muscle adaptation during energetic challenges.

## Results

### REDD1 deletion impairs metabolic adaptation under stress

We previously demonstrated that REDD1 over-expression following glucocorticoid (DEX) treatment or plasmid electroporation promotes skeletal muscle atrophy [32, 35]. Interestingly, REDD1 expression increases in response to hypoxia and fasting [35, 42], two energetic stresses known to promote amyotrophy [36, 37]. We hypothesize that REDD1 could also be responsible for skeletal muscle atrophy during hypoxia and fasting.

**Fig. 1:**
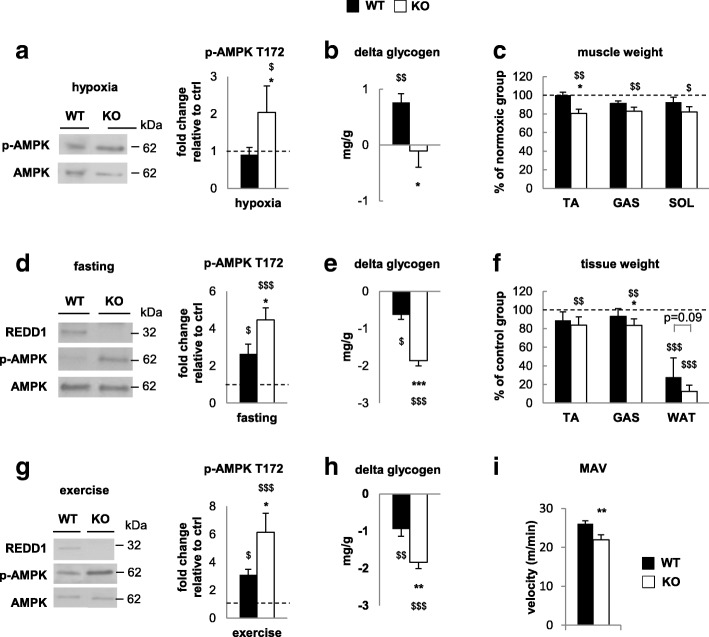
REDD1 deletion exacerbates energetic stress. **a** AMPK phosphorylation, **b** glycogen content, and **c** muscle weight of gastrocnemius (GAS), tibialis anterior (TA), and soleus (SOL) in 6-month-old WT and REDD1 KO mice exposed to 2 weeks of hypobaric hypoxia (6500 m) relative to normoxic controls (*n* = 7 per group, except for **b** KO *n* = 6). **d** AMPK phosphorylation, **e** glycogen content, and **f** weight of gastrocnemius (GAS), tibialis anterior (TA), and perigonadal white adipose tissue (WAT) in 6-month-old WT and REDD1 KO mice in response to food deprivation for 16 h (**d** and **e**) or 48 h (**f**) relative to fed controls (*n* = 8 per group except for **f** WT *n* = 6 and KO *n* = 7). **g** AMPK phosphorylation (*n* = 6 per group) and **h** glycogen content (WT *n* = 8 and KO *n* = 7) in skeletal muscle after a 90-min running exercise by 6-month-old WT and REDD1 KO mice relative to sedentary controls. **i** Maximum aerobic velocity (MAV) in 6-month-old WT and REDD1 KO mice (*n* = 11 per group). For clarity, we presented here only data normalized to their respective controls. All non-normalized values are available in Additional file 2: Figure S1. *p < 0.05, **p < 0.01, and ***p < 0.001 vs. corresponding WT group, and $*p* < 0.05, $$*p* < 0.01, and $$$*p* < 0.001 vs. corresponding to control group (same genotype) by two-way ANOVA and Fisher post-hoc test (**a–h**) or unpaired *t*-test (**i**). GAS gastrocnemius, KO knockout, MAV maximum aerobic velocity, SOL soleus, TA tibialis anterior, WAT white adipose tissue, WT wild type

Six-month-old wild-type (WT) and REDD1 KO mice were exposed to hypobaric hypoxia simulating an altitude of 6500 m for 2 weeks. Classical markers of hypoxic exposure, such as a reduction of food intake, heart hypertrophy, and an increase in hematocrit, were found in both REDD1 KO and WT mice after hypoxia (Additional file 1: Table S1). Contrary to our expectations, REDD1 deletion promoted skeletal muscle loss in response to hypoxia (Fig. 1c; Additional file 2: Figure S1 shows the full raw data used for Fig. 1) associated with the induction of genes involved in muscle proteolysis (FOXO1, BNIP3, Gabarapl, and LAMP2A; Additional file 2: Figure S2) in hypoxic REDD1 KO muscles only. Hypoxia is also known to increase production of reactive oxygen species (ROS) [43], and excessive ROS generation can lead to skeletal muscle atrophy [44]. Previous studies stated that REDD1 could alter ROS production [11, 30, 39]. Here, we did not observe marked differences between hypoxic WT and REDD1 KO mice regarding antioxidant enzymatic activities or gene expression and oxidized proteins (Additional file 2: Figure S3). Interestingly, muscles from REDD1 KO mice but not from WT mice displayed increased activation of the energetic stress sensor AMPK after 14 days of hypoxia (Fig. 1a), implying that REDD1 contributes to metabolic adaptation during hypoxia. Glycogen depletion is another marker of energetic stress. However, chronic hypoxia has been shown to result in higher muscle glycogen stores despite the greater utilization of carbohydrates [45]. Consistently, an elevated muscle glycogen content was found in hypoxic WT mice, while REDD1 KO mice failed to make this adaptation (Fig. 1b). Altogether, these data strongly suggest that REDD1 deletion exacerbates energetic stress under hypoxia leading to skeletal muscle atrophy. Similarly, we observed a greater atrophy in REDD1 KO muscles after 16-h fasting (Fig 1f) despite a higher mTORC1-dependent phosphorylation of S6, ULK1, and 4E-BP1 compared to WT mice (Additional file 2: Figure S4a). As seen under hypoxia, this marked muscle atrophy was associated with an increase of energetic stress assessed by glycogen depletion and AMPK activation (Fig. 1d and e). Altogether, these results support that REDD1 is necessary for skeletal muscle adaptation during energetic stresses such as hypoxia and fasting.

To confirm this novel hypothesis further, we challenged REDD1 KO mice with an endurance exercise, another energetic stress condition known to induce REDD1 expression [3]. Once again, REDD1 deletion exacerbated glycogen depletion and AMPK activation in response to exercise (Fig. 1g and h). The higher energetic stress experienced by REDD1 KO mice was also supported by the decrease in aerobic exercise performance compared to WT mice (Fig. 1i). Therefore, REDD1 deletion exacerbates energetic stress leading to skeletal muscle atrophy in hypoxia and fasting and impairment of exercise performance.

### REDD1 reduces O_2_ consumption in skeletal muscle

The exacerbation of energetic stress evidenced in REDD1-depleted conditions could be explained by two non-exclusive hypotheses: (i) a deficiency in ATP production or (ii) an elevation of energy demand. Interestingly, REDD1 has been proposed to localize in the mitochondria in vitro [11] and recent studies showed that REDD1 deletion in MEFs leads to alteration of mitochondrial function [38, 39]. Therefore, we hypothesized that the increase in energetic stress observed in REDD1 KO mice during metabolic challenges could be due to an alteration of mitochondrial function in skeletal muscle.

**Fig. 2:**
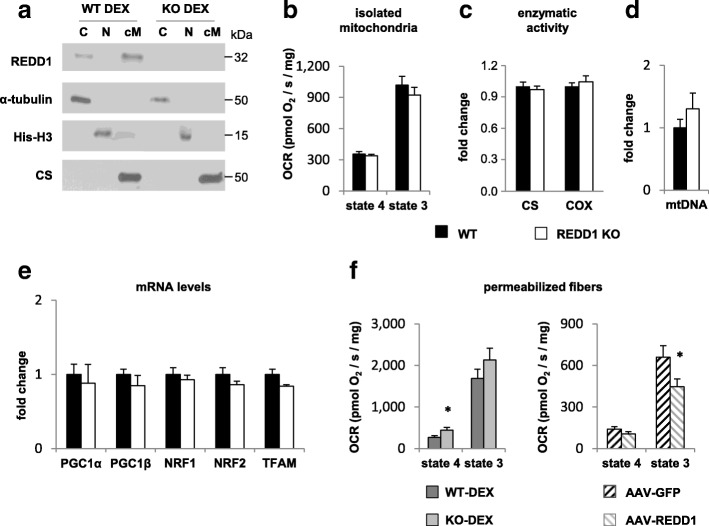
REDD1 reduces O_2_ consumption in skeletal muscle. **a** GAS skeletal muscle subcellular fractionation in REDD1 KO and WT mice 5 h after DEX treatment. The quality of fractionation was checked by detecting proteins specifically localized in each fraction: α-tubulin for the cytosol, histone-H3 (his-H3) for the nucleus, and citrate synthase (CS) for mitochondria. Mitochondria purity was also observed by transmission electron microscopy (Additional file 2: Figure S8). **b** O_2_ consumption rate (OCR) and respiratory control ratio (i.e., state 3 to state 4 ratio) of isolated mitochondria from skeletal muscles (GAS, QUAD, and TA) in the presence of lipidic substrates (palmitoylcarnitine + malate) in WT (*n* = 8) and REDD1 KO mice (*n* = 10). **c** Citrate synthase and cytochrome C oxidase (COX) basal activity in GAS muscle of WT and REDD1 KO mice (*n* = 7 per group). **d** Mitochondrial DNA (WT *n* = 5 and KO *n* = 6) and **e** expression of genes involved in mitochondrial biogenesis in WT (*n* = 7) and REDD1 KO mice (*n* = 6). **f** O_2_ consumption rate of permeabilized fibers from TA of WT and REDD1 KO mice treated with dexamethasone (DEX; *n* = 6 per group) and WT mice injected with AAV6 vectors encoding green fluorescent protein (GFP) or murine REDD1 (*n* = 12 per group). *p < 0.05 vs. corresponding control group by unpaired *t*-test. C cytosol, cM crude mitochondria, COX cytochrome C oxidase, CS citrate synthase, DEX dexamethasone, GAS gastrocnemius, GFP green fluorescent protein, his-H3 histone-H3, KO knockout, mtDNA mitochondrial DNA, N nucleus, NRF nuclear respiratory factor, OCR O_2_ consumption rate, PGC peroxisome proliferator-activated receptor gamma coactivator, QUAD quadriceps, TA tibialis anterior, TFAM mitochondrial transcription factor A, WT wild type

We first investigate whether REDD1 could localize in mitochondria in skeletal muscle. Since REDD1 protein is barely detectable in resting skeletal muscle [32, 42, 46], and that glucocorticoids, a component of energy stress, are responsible for REDD1 expression during fasting and exercise [42, 47], we used DEX treatment to induce strong endogenous REDD1 expression allowing mechanistic investigations. Following fractionation of fresh skeletal muscles, we observed that endogenous REDD1 protein is partly localized in a crude mitochondrial fraction after DEX treatment (Fig. 2a). Likewise, we observed mitochondrial localization of REDD1 in skeletal muscle after exercise (Additional file 2: Figure S5), suggesting that this subcellular localization occurs in various conditions. We then assessed the O_2_ consumption (VO_2_) of mitochondria isolated from WT and REDD1 KO skeletal muscles. No differences in VO_2_ were evidenced between WT and KO mice with ADP either coupled (state 3) or uncoupled (state 4) with either lipids (Fig. 2b) or carbohydrates as substrates (Fig. 2b and Additional file 2: Figure S6). Similarly, respiration of mitochondria isolated from skeletal muscles remained unchanged between WT and KO after REDD1 expression induction by DEX treatment (Additional file 2: Figure S6). Moreover, we did not find any difference between muscles from WT and REDD1 KO mice for citrate synthase and cytochrome oxidase activities (Fig. 2c), mitochondrial DNA content (Fig. 2d), or expression of genes involved in mitochondrial biogenesis (Fig. 2e). These results show that REDD1 deletion did not alter the mitochondrial content or respiration of mitochondria isolated from skeletal muscle.

In contrast, by performing similar respiration experiments using permeabilized skeletal muscle fibers instead of isolated mitochondria, we observed an increase in VO_2_ for REDD1 KO compared to WT mice (Fig. 2f). To confirm further that REDD1 directly acts on VO_2_ in skeletal muscle fibers, we overexpressed REDD1 in WT mice using an AAV. As expected, the intramuscular injection of AAV-REDD1 decreased O_2_ consumption in WT permeabilized fibers (Fig. 2f) without any change in mitochondrial content (based on citrate synthase activity; Additional file 2: Figure S7). Since no alteration was evidenced with isolated mitochondria, this result suggests that the cellular context is important to reveal the effect of REDD1 on mitochondrial respiration. Collectively, our results indicate that REDD1 may regulate the connection of mitochondria with other adjacent organelles like the ER, rather than directly affecting mitochondrial intrinsic respiration.

### REDD1 disrupts the mitochondria/ER interaction

**Fig. 3:**
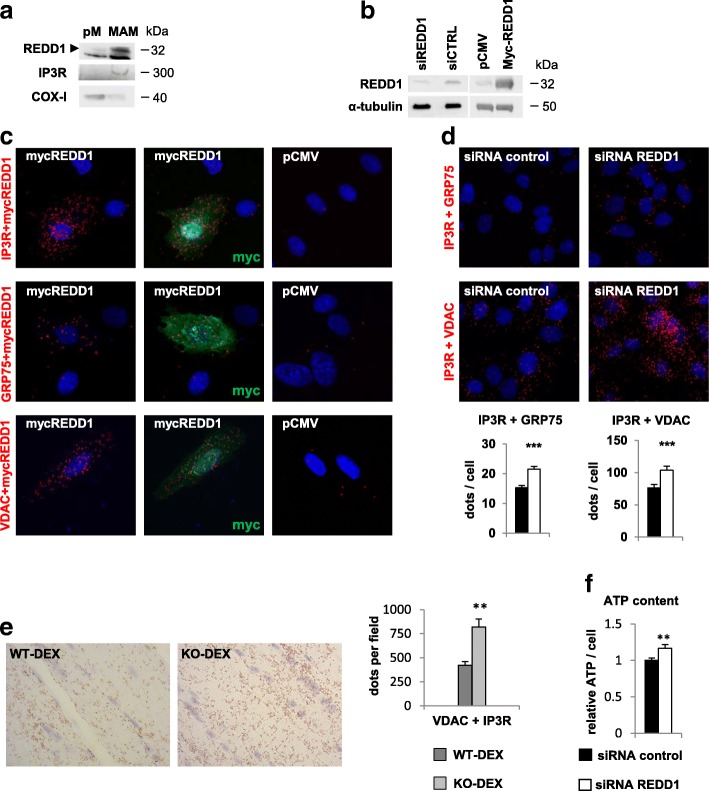
REDD1 interacts with and modulates the extent of MAMs. **a** Pure mitochondria (pM) and MAMs fraction obtained from WT mice skeletal muscle 5 h after DEX treatment (1 mg/kg). Inositol trisphosphate receptor (IP3R) and cytochrome C oxidase-I (COX-I) are specific to MAMs and the mitochondria compartments, respectively. **b** Representative blots for REDD1 knockdown by siRNA or REDD1 overexpression in human myoblasts. **c** Proximity ligation assay between IP3R and REDD1, GRP75 and REDD1, or VDAC and REDD1 in human myoblasts transfected with either an empty vector (pCMV) or a plasmid encoding murine REDD1 fused to Myc (mycREDD1). **d** Representative pictures and corresponding quantifications of proximity ligation assay between GRP75 and IP3R or VDAC and IP3R with either control siRNA or siRNA against REDD1 in human primary myoblasts (*n* = 6 per condition from three separate cultures). **e** Representative images and quantitative analysis of the VDAC1/IP3R1 interactions measured by in situ proximity ligation assay in paraffin-embedded muscle from WT (*n* = 5) and REDD1 KO mice (*n* = 6) treated for 5 h with DEX. **f** ATP content measured by luciferase assay in human primary myoblasts transfected with either control siRNA (*n* = 10 from three separate cultures) or siRNA REDD1 (*n* = 11 from three separate cultures). **p < 0.01 and ***p < 0.001 vs. control by unpaired *t*-test. COX cytochrome C oxidase, DEX dexamethasone, IP3R inositol trisphosphate receptor, KO knockout, MAMs mitochondrial-associated endoplasmic reticulum membranes, pM pure mitochondria, VDAC voltage-dependent anion channel, WT wild type

The ER/mitochondria crosstalk promotes mitochondrial respiration through the formation of a specific membrane interaction known as MAMs. Interestingly, we showed that REDD1 is localized in crude mitochondria (Fig. 2a), a fraction containing both pure mitochondria and associated membranes (Additional file 2: Figure S8a). By further fractionation of the crude mitochondria, we demonstrated that REDD1 is not present in pure mitochondria but specifically in the mitochondria/ER interaction domain (Fig. 3a). As both MAMs formation [20] and REDD1 deletion increase VO_2_ (Fig. 2f), we hypothesized that REDD1 could impair MAMs integrity. We confirmed that REDD1 localizes in MAMs in human primary myoblasts by showing that overexpressed REDD1 directly binds to the voltage-dependent anion channel (VDAC), 75 kDa glucose-regulated protein (GRP75), and IP3R, three proteins of the calcium channeling complex localized in the MAMs fraction (Fig. 3b,c) [20, 48]. In MAMs, the IP3R/GRP75/VDAC protein complex physically links mitochondria and ER [20, 48], and quantification by a proximity ligation assay (PLA) of IP3R/VDAC, GRP75/IP3R or GRP75/VDAC protein interactions was used to assess the extent of MAMs [48]. We showed here in human primary myoblasts by in situ PLA that silencing by small interfering RNA (siRNA) of REDD1 significantly increased the protein complexes IP3R/VDAC (+ 36%) and IP3R/GRP75 (+41%; Fig. 3b, d) as well as the ATP concentration (Fig. 3f). Importantly, we also demonstrated in vivo that REDD1 deletion results in a strong increase in the amount of MAMs (+90%) in skeletal muscle of DEX-treated mice assessed via the VDAC/IP3R complex (Fig. 3e). Altogether, these results indicate that REDD1 modulates the extent of MAMs and basal energetic metabolism in skeletal muscle cells via structural disruption of the mitochondria/ER interface. The effect of REDD1 on mitochondrial respiration and ATP content could, therefore, originate from its action on MAMs.

### REDD1 inhibits the Akt/mTOR pathway in the mitochondria/ER interface

**Fig. 4:**
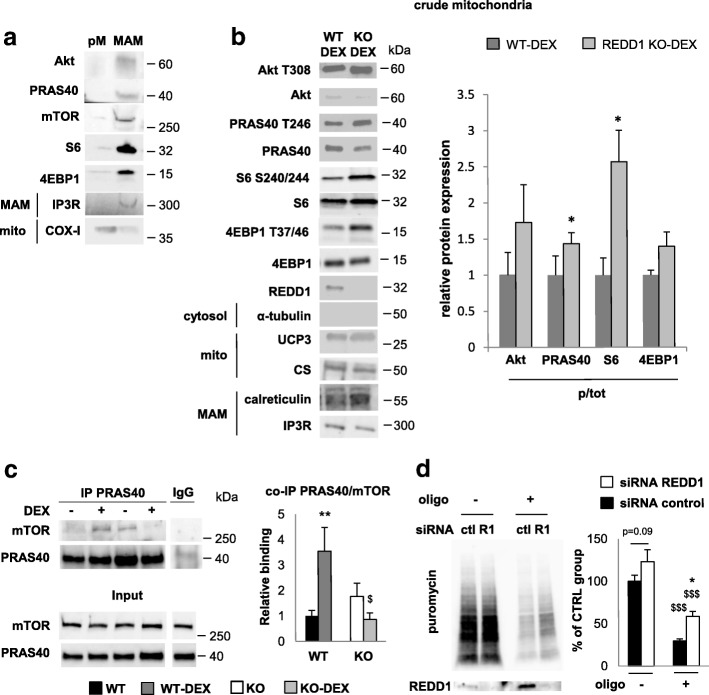
REDD1 inhibits the Akt/mTOR pathway in the mitochondria/ER interface. **a** Pure mitochondria (pM) and MAMs fraction obtained from skeletal muscle of WT mice 5 h after DEX treatment (1 mg/kg). The quality of fractionation was checked by detecting proteins specifically localized in each fraction (IP3R for MAMs and COX-I for mitochondria). **b** Crude mitochondrial protein expression in WT and REDD1 KO mice acutely treated with DEX (1 mg/kg; *n* = 3 per group except for S6 WT, 4EBP1 WT, and KO: *n* = 4). CS and uncoupling protein 3 (UCP3) were used as markers for mitochondria (mito). The presence of MAMs was checked by calreticulin and IP3R expression. **c** mTORC and PRAS40 association in crude mitochondrial lysate of WT and REDD1 KO mice acutely treated with DEX (1 mg/kg; WT *n* = 7, WT-DEX *n* = 6, KO *n* = 3, and KO-DEX *n* = 4). **d** Representative blots for puromycin incorporation and corresponding quantification in human myoblasts treated with either siRNA control (ctl) or siRNA directed against REDD1 (R1) after 1 h of glucose deprivation and treatment with the ATP synthase inhibitor oligomycin (2 μmol/l; *n* = 3 per group from three separate cultures). *p < 0.05 vs. corresponding control. $$$*p* < 0.001 vs. oligomycin-treated cells by unpaired *t*-test (**b**) or two-way ANOVA and Fisher post-hoc test (**c, d**). COX cytochrome C oxidase, CS citrate synthase, ctl control, DEX dexamethasone, IP3R inositol trisphosphate receptor, KO knockout, MAMs mitochondrial-associated endoplasmic reticulum membranes, mito mitochondria, oligo oligomycin, pM pure mitochondria, p/tot phospho-to-total ratio, R1 REDD1, UCP3 uncoupling protein 3, WT wild type

Whilst ATP production and mitochondrial integrity were not impaired by REDD1 deletion (Figs. 2 and 3), REDD1 expression disrupts MAMs (Fig. 3), which are important in the coupling between energy production and anabolic processes [49]. Therefore, REDD1 deletion could lead to an inappropriate activation of MAMs-dependent anabolic processes. We suspected that the exacerbated energetic stress observed in REDD1 KO mice during a metabolic challenge was caused by an increase in energy demand due to an excessive activation of the Akt/mTOR pathway. Indeed, MAMs and the Akt/mTOR signaling pathway are closely interconnected. On one hand, MAMs are required for Akt activation by growth factor [48], and on the other hand, Akt/mTOR activation increases the mitochondria/ER interaction [19]. Furthermore, the Akt/mTOR pathway promotes ATP-demanding processes, such as protein synthesis [15], in the ER. Interestingly, REDD1 is known to inhibit Akt/mTOR-dependent protein synthesis in skeletal muscle via PRAS40 [32], and we detected several proteins of this signaling pathway (Akt, PRAS40, and mTOR) in a purified MAMs fraction but not in pure mitochondria (Fig. 4a). We also checked for the presence of VDAC (a MAMs protein localized on the outer membrane of the mitochondria), mTOR, and PRAS40 in crude mitochondria by immunofluorescence staining (Additional file 2: Figure S8b).

To assess whether REDD1 could inhibit the Akt/mTOR pathway specifically in MAMs, we analyzed the phosphorylation status of Akt, PRAS40, and mTOR targets (S6 and 4EBP1) in the crude mitochondria fraction (containing MAMs) isolated from fresh muscles. We observed that REDD1 deletion significantly increased PRAS40 phosphorylation (Fig. 4b). As dephosphorylated PRAS40 is known to inhibit mTORC1 by direct binding [27], we predicted that PRAS40 could also interact with mTOR in this same subcellular compartment. In agreement, endogenous PRAS40 and mTOR co-precipitated in the crude mitochondrial lysate, and this association was strongly increased following REDD1 expression upon DEX treatment in WT but not in REDD1 KO mice (Fig. 4c). Moreover, PRAS40 over-phosphorylation in REDD1 KO mice was associated with a greater phosphorylation of the mTORC1 downstream target S6 in the crude mitochondria fraction (Fig. 4b). This result shows that REDD1 is necessary for inhibiting mTOR activity in MAMs.

To evaluate the impact on protein translation, we monitored puromycin incorporation in human myoblasts under energetic challenging conditions (1 h glucose depletion and ATP synthase inhibition with oligomycin). In these conditions, REDD1 silencing led to an increase in protein synthesis (Fig. 4d). This negative effect of REDD1 on translation appears, however, blunted in the presence of glucose, likely because cells rely mainly on glycolysis rather than on mitochondria to produce ATP (Additional file 2: Figure S9).

mTOR activity has been correlated with energy expenditure measured by basal VO_2_ [23] or ATP depletion [25] and we confirmed here that inhibition of mTOR catalytic activity by Torin1 treatment reduced basal VO_2_ in myoblasts (Additional file 2: Figure S10a). Therefore, REDD1 contributes to reducing VO_2_ by regulating an Akt/PRAS40/mTOR signaling pathway at the mitochondrial/ER interface and decreasing protein synthesis. The increase in energetic stress during metabolic challenges in REDD1 KO mice could be explained by an inappropriate activation of the Akt/mTOR pathway consistently with the higher activation of the Akt/mTOR pathway found in REDD1 KO mice following exercise or fasting (Additional file 2: Figure S4a, b).

### REDD1 limits HKII mitochondrial recruitment

**Fig. 5:**
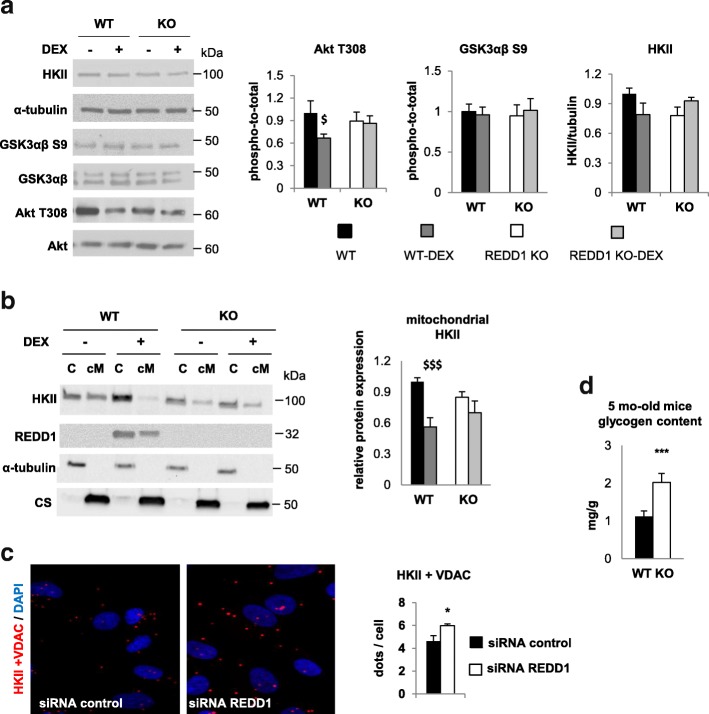
REDD1 controls Hexokinase II (HKII) mitochondrial localization. **a** Akt T308 (WT *n* = 8, WT-DEX *n* = 9, KO *n* = 7, and KO-DEX *n* = 8), GSK3 S9 (WT and KO *n* = 8, and WT-DEX and KO-DEX *n* = 7) and HKII (WT, WT-DEX, and KO-DEX *n* = 7 and KO *n* = 8) protein expression in total lysate and **b** cytosolic (C) and crude mitochondrial (cM) subcellular localization of HKII in WT and REDD1 KO mice acutely treated with 1 mg/kg of dexamethasone (DEX; WT *n* = 7, WT-DEX *n* = 6, KO *n* = 3, and KO-DEX *n* = 4). **c** Representative pictures and corresponding quantifications of proximity ligation assay between VDAC and HKII with either control siRNA or siRNA against REDD1 in human primary myoblasts (*n* = 7 per condition from one culture). **d** Glycogen content in gastrocnemius of WT (*n* = 11) and REDD1 KO mice (*n* = 13). $*p* < 0.05 and $$$*p* < 0.001 vs. corresponding untreated group by two-way ANOVA and Fisher post-hoc test (**a, b**). *p < 0.05 and ***p < 0.001 vs. WT or control siRNA by unpaired *t*-test (**c, d**). C cytosol, cM crude mitochondria, CS citrate synthase, DEX dexamethasone, KO knockout, VDAC voltage-dependent anion channel, WT wild type

In addition to its role in muscle protein synthesis, phosphorylated Akt enables glycogen storage [16, 17] through glucose uptake [50], HKII activation by mitochondrial recruitment [51], and inhibition of the glycogen synthase repressor GSK3 [28]. This notably requires glucose phosphorylation by mitochondrial HKII through an ATP-dependent reaction. Importantly, HKII has also been shown to localize in mitochondria through direct interaction with VDAC [19]. We sought to determine whether REDD1 could alter HKII localization and glycogen content. Indeed, REDD1 silencing in human primary myoblasts resulted in an enhanced VDAC/HKII interaction (+30%), as assessed by PLA (Fig. 5c). Conversely, REDD1 induction by DEX reduced both mitochondrial HKII (Fig. 5b) and Akt phosphorylation on T308 in skeletal muscle (Fig. 5a). Moreover, the glycogen content in the basal state was significantly higher in skeletal muscles from REDD1 KO mice compared to WT controls (Fig. 5d), despite there being no change in GSK3 phosphorylation in the presence of REDD1 (Fig. 5a). Altogether, this strongly suggests that REDD1 inhibits glycogen storage via restriction of mitochondrial HKII localization. Additionally, we confirmed that mitochondrial HKII activity contributes to increase VO_2_ in myoblasts (Additional file 2: Figure S10b), as previously shown by others [52]. This shows that REDD1-mediated inhibition of HKII also participates in the reduction in O_2_ consumption and energy demand.

### REDD1 reduces basal metabolism in vivo

**Fig. 6:**
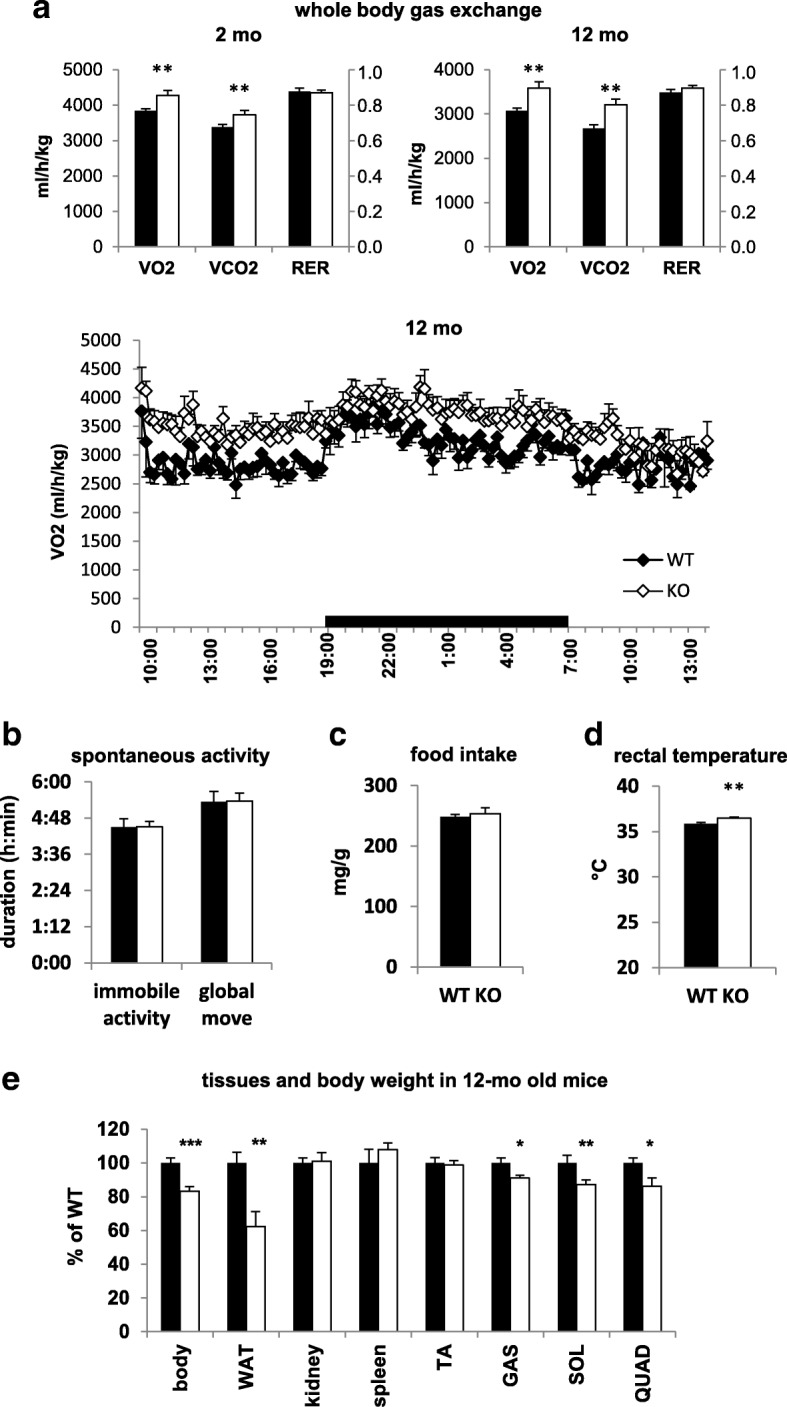
REDD1 deletion increases basal metabolism in vivo. **a** Basal whole-body O_2_ and CO_2_ fluxes in young (2 months) and adult mice (12 months; *n* = 6 per group). **b** Global and immobile voluntary activity (*n* = 14 per group). **c** Food intake (*n* = 14 per group). **d** Rectal temperature measured at 22 °C in 5–6-month-old WT (*n* = 8) and KO mice (*n* = 9). **e** Body (*n* = 10 per group), white adipose tissue (WAT; WT *n* = 8 and KO *n* = 7), kidney (WT *n* = 6 and KO *n* = 7), spleen (*n* = 6 per group), tibialis anterior muscle (TA; *n* = 8 per group), gastrocnemius muscle (GAS; *n* = 8 per group), soleus muscle (SOL; WT *n* = 10 and KO *n* = 9), and quadriceps muscle (QUAD; WT *n* = 7 and KO *n* = 6) weight in 12–13-month-old WT and REDD1 KO mice. *p < 0.05, **p < 0.01 and ***p < 0.001 vs. corresponding WT group by unpaired *t*-test. Black bars are CTRL WT and open bars are CTRL KO. CTRL control, GAS gastrocnemius, KO knockout, mo month, QUAD quadriceps, RER respiratory exchange ratio, SOL soleus, TA tibialis anterior, WAT white adipose tissue, WT wild type

Skeletal muscle accounts for the greatest part of the basal metabolism rate (around 30%) [53]. Considering the role of REDD1 in the regulation of the energy expenditure that is dependent on anabolic processes of skeletal muscle, we expected that REDD1 KO mice should have a hypermetabolic profile. Therefore, we looked to see whether REDD1 deletion would affect whole-body metabolism. We observed a significant increase in O_2_ consumption and in CO_2_ production in both young (2 months) and adult (12 months) REDD1 KO mice in comparison to WT (Fig. 6a), in agreement with data obtained for native REDD1 KO MEFs [38]. Importantly, these differences are not due to a shift in the nature of the oxidized substrates (unchanged respiratory exchange ratio; Fig. 6a), enhanced spontaneous activity (Fig. 6b), or modified food intake (Fig. 6c). The increase in basal metabolism rate is further supported by a greater rectal temperature in REDD1 KO mice compared to WT mice (Fig. 6d). Moreover, while there was no difference in skeletal muscle mass and body weight between young REDD1 KO and WT mice [6, 32, 54], 12- to 13-month-old REDD1 KO mice displayed a reduction in body weight, skeletal muscle mass, and abdominal white adipose tissue deposit compared to WT (Fig. 6e). This indicates a depletion of metabolic substrate stores (of proteins and lipids) in old REDD1 KO mice. Altogether, our results show that REDD1 reduces basal metabolism.

## Discussion

We have previously shown that REDD1 is important in mediating muscle atrophy caused by chronic glucocorticoid administration [32]. Unexpectedly, we observed in this report that under physiological energetic stress (hypoxia, fasting, or exercise), REDD1’s function in mice is to spare muscle mass and to reduce energy expenditure. This led us to explore the role played by REDD1 in skeletal muscle during these energetically challenging conditions.

### REDD1 reduces energetic metabolism and MAMs extent

Our results unveil a metabolic role of REDD1 as a gatekeeper of Akt/mTORC1-mediated synthesis processes leading to a decrease in O_2_ and ATP consumption. In agreement with data obtained by the group of Lafarge et al. for primary REDD1 KO MEFs [38], we observed higher whole-body O_2_ consumption in REDD1 KO mice. Consistently, we showed that REDD1 overexpression reduces O_2_ consumption in permeabilized fibers prepared from fresh muscles. Interestingly, we did not observe any effect due to REDD1 overexpression or deletion using isolated mitochondria, suggesting that REDD1 could modulate the mitochondrial crosstalk with other cellular organelles or structures such as MAMs, rather than playing a direct role in mitochondrial activity per se. Indeed, we determined here that REDD1 localizes in the MAMs fraction (and not in pure mitochondria) isolated from the skeletal muscles of mice treated with glucocorticoids. Importantly, we demonstrate that REDD1 inhibition resulted in a greater MAMs extent, in cultured human myoblasts and in mice skeletal muscles. Since MAMs promote mitochondrial respiration [20], our results strongly suggest that REDD1 decreases basal metabolism through inhibition of MAMs formation in response to glucocorticoids, a major component of energetic stress adaptation.

**Fig. 7:**
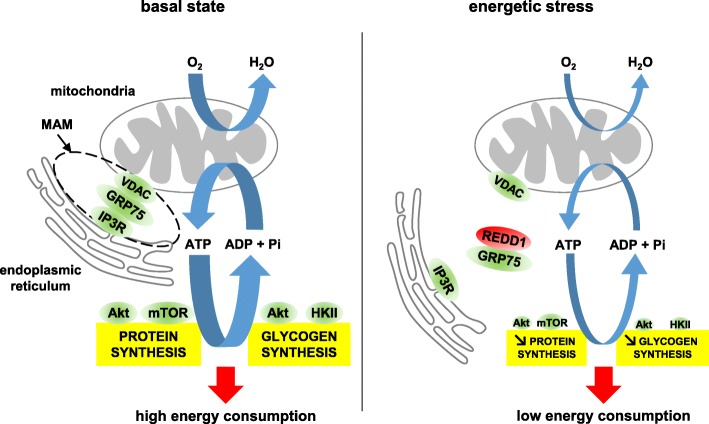
REDD1 controls stress-induced reduction in O_2_ consumption and anabolic processes. In the basal state, activation of the Akt/mTOR pathway promotes (i) anabolic processes (protein and glycogen synthesis), (ii) the mitochondria/ER interaction, and (iii) oxygen-dependent ATP production. In response to energetic stress, REDD1 inhibits the Akt/mTOR pathway and interacts with MAMs, leading to a coordinated decrease in MAMs extent, mitochondrial oxygen consumption, and anabolic processes. This mechanism could contribute to spare ATP in stressful conditions

As impairment of Akt/mTOR signaling has been shown to disrupt MAMs [19, 55], REDD1 could thus reduce MAMs extent through inhibition of Akt/mTOR in this specific compartment. The activation of mTORC1 is associated with an increase in O_2_ and ATP consumption [23, 25]. Supporting this, treatment with the mTORC1 inhibitor rapamycin decreases kidney, liver, cardiac, and whole-body O_2_ consumption [56, 57] and preserves the neural ATP level [25]. Akt/mTORC1 activation is related to anabolic processes (i.e., protein translation and glycogen synthesis) driven by this pathway. We found here a REDD1/PRAS40-dependent inhibition of TORC1 at the mitochondrial/ER interface. It is, thus, tempting to propose that MAMs disruption by REDD1 inhibits protein synthesis by reducing the ATP supply to ER in addition to the repression of mTORC1-dependent messenger RNA (mRNA) translation initiation (Fig. 7).

Moreover, we found from a mass spectrometry analysis that endogenous REDD1 and GRP75, a MAM protein bridging ER and the outer mitochondrial membrane, can be co-immunoprecipitated in mice skeletal muscle lysates (data not shown). This suggests that REDD1 may also affect MAMs formation via direct GRP75 binding. Further work is needed to describe fully how REDD1 reduces the extent of MAMs and to identify the molecular mechanisms.

### Is REDD1 required for autophagy or mitophagy?

Conflicting results on mitochondrial respiration in REDD1-depleted MEFs have been recently published. On one hand, Lafarge et al. show that REDD1-depleted MEFs had an increased respiration rate, as we observed here for permeabilized myofibers [38]. On the other hand, Qiao et al. reported the reverse outcome in which REDD1-depleted immortalized MEFs exhibit decreased O_2_ consumption. These authors proposed that REDD1 inhibition impairs mitophagy leading to an accumulation of defective mitochondria [39]. However, we did not find any marker of either defective mitochondria or mitochondrial accumulation in muscles of REDD1 KO mice (citrate synthase and cytochrome c oxidase activities, mitochondrial DNA content, and respiration of isolated mitochondria were all unchanged). Our results indicate that REDD1 appears dispensable for autophagy and mitophagy in skeletal muscle after intensive endurance exercise (assessed by mitochondrial DNA content, and BNIP3 and ULK S317 levels; Additional file 2: Figure S11) as well as after hypoxia (BNIP3, Gabarapl and LAMP2A mRNA expression; Additional file 2: Figure S2). Moreover, it has recently been shown that stimulating OXPHOS enhances mitophagy to facilitate mitochondrial renewal [58]. Incidentally, the raised mitochondrial respiration we observed in REDD1 KO myofibers further supports the higher mitophagy observed in REDD1 KO mice. However, a recent study shows that REDD1 deletion in vivo impairs mitophagy in mice cartilage [59], suggesting that REDD1’s function could be tissue dependent. Future experiments should help to clarify the role played by REDD1 in autophagy and mitophagy in different cellular contexts.

### REDD1 deletion exacerbates energetic stress

We highlight here that the increase in energy and O_2_ demand in REDD1 KO mice results in a maladaptation to energetic stresses. In fact, upon various energetic challenges (hypoxia, fasting, and exercise), we observed an over-activation of AMPK in the absence of REDD1. Our data agree with a recent study showing an increase in AMPK phosphorylation and ATP depletion in REDD1 KO primary chondrocytes during energetic stress [59]. Parallel to AMPK over-activation, we found greater glycogen depletion after a running exercise or fasting in REDD1 KO compared to WT muscles. This suggests that the fuel requirement is exacerbated in REDD1 KO mice to cope with stress (food deprivation, O_2_ reduction, or increase in ATP demand). Several studies have shown that activation of Akt increases glycogen content, energy expenditure, and basal VO_2_ in mice [18, 60, 61]. Glycogen storage depends upon glucose-6-phosphate availability and Akt controls the formation of glucose-6-phosphate, by regulating the mitochondrial localization and activation of HKII [29]. Our results indicate that REDD1 interferes with this cascade of events by inhibiting HKII targeting to mitochondria, therefore limiting glycogen storage and energy demand. In fact, we recorded a higher basal glycogen content in REDD1 KO mice. Thus, in a fed state, REDD1 deletion fosters glycogen storage through over-activation of the Akt/HKII pathway. In contrast, during energetic stress, REDD1 deletion leads to an inappropriate activation of anabolic processes, resulting in increased energy demand and glycogen depletion.

Interestingly, Reiling and Hafen showed that REDD1 overexpression contributed to extend the life span of *Drosophila* under starvation conditions [62], underlining the crucial role of REDD1 in adaptation to nutrient deprivation. Moreover, we observed here that REDD1 KO mice display impaired aerobic performance, further demonstrating that REDD1 deficiency disrupts energetic metabolism during exercise. We verified that the hypermetabolic phenotype of REDD1 KO mice (greater O_2_ consumption and rectal temperature) was not due to a difference in food intake, confirming data reported by others [46]. Chronic elevated energy expenditure leads to a metabolic imbalance that would ultimately result in reduced storage of macronutrients, as we observed in 12-month-old REDD1 KO mice. Altogether, these data reinforce that REDD1’s function is indispensable for skeletal muscle adaptation to energetic stresses and they raise the issue of the role of REDD1 in energetic metabolism homeostasis during aging.

### Regulation of muscle mass by REDD1

We previously demonstrated that REDD1 deficiency is protective against skeletal muscle atrophy following glucocorticoid treatment [32]. In contrast, we show here that REDD1 deletion rather exacerbates hypoxia-mediated and fasting-mediated amyotrophy. A likely explanation of this apparent paradoxical effect of REDD1 is that fuel substrates for ATP and macromolecule synthesis are not limiting during glucocorticoid treatment. In this case, sustained Akt/mTORC1-dependent anabolism with REDD1 deletion has no detrimental effects on energetic status and muscle mass. In contrast, during energetic stress (hypoxia and fasting), the limited down-regulation of the Akt/mTORC1 pathway due to the absence of REDD1 will disturb the energy balance (expenditure vs. production). This worsens the metabolic crisis, ultimately resulting in exacerbated muscle wasting. In agreement, we recorded AMPK overactivation in REDD1 KO muscle during energetic stress and AMPK is known to promote muscle protein breakdown through the FOXO-dependent atrophying program [63]. Consistently, we observed increased expression of genes involved in the ubiquitin-proteasome system or in autophagy in hypoxic REDD1-deficient mice.

We propose that REDD1 acts as a rheostat of AMPK activation by limiting an energetic crisis and lowering the energy expenditure caused by unrestrained Akt/mTORC1 signaling, which ultimately restricts the loss of muscle mass. Therefore, trying to maintain the Akt/mTOR-dependent protein synthesis during an amyotrophic process that is secondary to an alteration of energy homeostasis does not appear to be an appropriate therapeutic strategy to spare skeletal muscle mass.

## Conclusions

In summary, we highlight a novel physiological metabolic role for REDD1 in glucocorticoid signaling, which enables the acclimatization of skeletal muscle during energetic challenges via the reduction of O_2_ and ATP consumption assigned to synthesis processes (Fig. 7). This could help our understanding of the pathologies involving REDD1, including cancer, diabetes, emphysema, depression, and Parkinson’s disease.

## Methods

### Animals

Whole-body REDD1 null mice were generated by Lexicon Genetics Inc. (The Woodlands, TX) specifically for Quark Pharmaceuticals Inc. (Fremont, CA) as previously described. They are the property of Quark Pharmaceuticals Inc. WT and KO mice were generated from C57Bl6 heterozygous × heterozygous crosses. Genotyping was performed from tail-derived genomic DNA as previously described [6]. Mice were housed in standard cages with free access to food and water under a 12-h dark–light cycle. All animals were killed by cervical dislocation and the tissues were harvested in the morning at the same time each day. These experiments were performed according to European directives (86/609/CEE) and approved by the national ethics committee (referral file APAFIS#9706-2,017,042,516,166,776 v3).

### Hypoxia experiment

WT and REDD1 KO mice were housed in a hypobaric chamber to simulate an altitude of 6500 m for 11 days after 3 days of progressive acclimatization. Tissues were harvested after cervical dislocation.

### Dexamethasone administration

WT and KO mice were treated with 1 mg/kg of DEX by oral gavage. Animals were sacrificed 5 h after gavage and their gastrocnemius and quadriceps muscles were removed for subsequent analyses.

### Exercise experiments

Maximum aerobic velocity was determined during a running exercise using a motorized treadmill. The speed was set to 10 m/min for 2 min, after which it was increased by 1 m/min every 60 s until exhaustion. An intensive exercise experiment was performed by setting the speed to 10 m/min for 60 min, after which it was increased by 1 m/min every 2 min for 30 min. Muscles were removed immediately at the end of the experiment period (90 min) after cervical dislocation and they were quickly frozen in liquid nitrogen and stored at −80 °C. Before each experiment (determination of maximum aerobic velocity or prolonged exercise), the mice were accustomed to the treadmill with a 5-min run at 10 m/min for 3 d, the last session occurring 48 h before the test.

### Western blotting

Sodium dodecyl sulphate-polyacrylamide gel electrophoresis (SDS-PAGE) and Western blots were performed as previously described [32]. Briefly, all antibodies were incubated on membranes overnight at 1:1000 except for α-tubulin (1:2000), mTOR, HK II, calreticulin, histone H3, ULK1, and phospho-ULK1 ser^757^ and ser^317^ (1:500) and IP3R (1:125). The next day, membranes were washed and incubated with HRP (Horseradish peroxidase)-conjugated secondary antibody at 1:3000 for 1 h at room temperature. Membranes were developed with Enhanced chemioluminescent (ECL) reagent using a Chemidoc™ Touch Imaging System (BioRad). A list of all antibodies is given in Additional file 1: Table S2.

### Skeletal muscle subcellular fractionation

Subcellular fractionation was performed on fresh muscles according to the method described by Dimauro et al. [64]. Briefly, the right and left gastrocnemius muscles were homogenized in STM buffer (250 mM sucrose, 50 mM Tris HCl (pH 7.4), 5 mM MgCl_2_, and a protease/phosphatase inhibitor cocktail). Muscle homogenate was centrifuged at 800*g* to pellet the nuclear fraction. The nuclear fraction was successively washed and centrifuged three times in STM buffer, then resuspended in NET buffer [20 mM HEPES (pH 7.9), 1.5 mM MgCl_2_, 0.5 M NaCl, 0.2 mM ethylenediaminetetraacetic acid (EDTA), 20% glycerol, 1% triton X100, and a protease/phosphatase inhibitor cocktail]. Other fractions were centrifuged at 800*g* to eliminate potentially contaminating nuclei still present in the homogenate. Then, the supernatant was centrifuged at 11,000*g* to separate the crude mitochondrial fraction (pellet) from the cytosolic and microsomal fraction (supernatant). The mitochondrial fraction was washed in STM buffer and centrifuged at 11,000*g* and finally resuspended in SOL buffer [50 mM Tris-HCl (pH 6.8), 1 mM EDTA, 0.5% triton X100, and a protease/phosphatase inhibitor cocktail] or CHAPS buffer for immunoprecipitation (0.3% CHAPS, 40 mM HEPES pH 7.5, 120 mM NaCl, 1 mM EDTA, 50 mM NaF, 1.5 mM Na_3_VO_4_, 10 mM β-glycerophosphate, and a protease inhibitor cocktail). The cytosolic and microsomal fraction was ultra-centrifuged at 100,000*g* to pellet the microsomes and other contaminant organelles. Cytosolic proteins in the supernatant were precipitated with acetone and centrifuged at 12,000*g*. Finally, the supernatant was discarded and the cytosolic proteins precipitated in the pellet were resuspended in STM buffer. MAMs fractions were isolated from the crude mitochondrial fraction as previously described [65].

### Immunoprecipitation of mitochondrial fraction

Gastrocnemius mitochondria were obtained by subcellular fractionation as explained above. In total, 500 μg of proteins from the lysate were diluted in a final volume of 500 μl with the CHAPS buffer. Precleared lysates were incubated with either 3 μg of anti-PRAS40 or 3 μg of anti-rabbit immunoglobulin G overnight at 4 °C. Samples were then incubated 1 h at 4 °C with 100 μl of 20% protein G sepharose beads (Cell Signaling Technology), collected by centrifugation, and washed four times with CHAPS buffer before analysis by western blot.

### Mitochondrial respiration

Gastrocnemius muscles were quickly excised and immediately placed into ice-cold buffer (100 mM KCl, 5 mM MgSO_4_, 5 mM EDTA, and 50 mM Tris-HCl, pH 7.4). Muscles were freed of connective tissues, minced, homogenized with an Ultra-turax homogenizer, and treated with Subtilisin A (0.1 mg/g wet muscle). Mitochondria were separated by centrifugation at 8000*g*, then at 800*g*. Finally, the mitochondria were pelleted from the supernatant at 9000*g*. The mitochondria were resuspended in 100 mM KCl and 10 mM MOPS (3-(N-Morpholino) propanesulfonic acid), pH 7.4. Mitochondria oxygen consumption was measured using the high-resolution Oxygraph-2 k (OROBOROS Instruments). Isolated mitochondria were incubated in two sealed thermostated chambers (37 °C) containing 2 ml of MIRO5 respiration medium (0.5 mM EGTA, 3 mM MgCl_2_.6H_2_O, 65 mM KCl, 20 mM taurine, 10 mM KH_2_PO_4_, 20 mM HEPES, 110 mM sucrose, and 1 g/l bovine serum albumin, pH 7.1). Resting rate (state 4) was evaluated in the presence of 2.5 mM malate, 5 mM glutamate, and 5 mM succinate or 40 μM palmitoylcarnitine and 2.5 mM malate. ADP-stimulated rate (state 3) was determined after addition of 0.5 mM ADP. Oxygen consumption was normalized to the total protein content of each sample measured using the Bradford assay.

For permeabilized fibers experiment, about ten pools of four muscle fibers were isolated from the tibialis anterior in ice-cold biopsy preservation solution (BIOPS: K_2_-EGTA 7.23 mM, CaK_2_-EGTA 2.77 mM, imidazole 20 mM, dithiothreitol 0.5 mM, KH_2_PO_4_ 3 mM, MgCl_2_ 6.56 mM, bovine serum albumin 0.2%, taurine 20 mM, ATP 5.3 mM, 2-(N-morpholino) ethane-sulfonic acid hydrate 53.3 mM, phosphocreatine 15 mM, pH 7.1). Fibers were then transferred into 1 ml of BIOPS containing 50 μg/ml of saponin for 30 min and washed twice in BIOPS free from saponin. The permeabilized fibers were introduced into an oxygraph chamber containing MIRO5 medium. Respiration was recorded after addition of the following substrates: pyruvate 5 mM, malate 5 mM, and ADP 5 mM. Oxygen consumption was normalized to the total protein content of each sample measured using the Bradford assay. Data acquisition and analysis were performed using Oxygraph-2 k-DatLab software version 4.3. The respiratory control ratio was calculated as the ratio of oxygen consumption at state 3 over oxygen consumption at state 4.

### Metabolic analysis

Respiratory gas exchanges were measured at 22 °C using a Comprehensive Lab Animal Monitoring System (Columbus Instruments) as previously described [66]. Mice were acclimatized individually in metabolic cages for 16 h before the 24 h recording. The respiratory exchange ratio was calculated as the ratio between CO_2_ production and O_2_ consumption.

### Glycogen content

Muscle glycogen content was measured as previously described [66]. Briefly, 30–50 mg of quadriceps muscle was dissolved in 30% KOH saturated with Na_2_SO_4_ at 100 °C for 20 min. Glycogen was precipitated by addition of 1.2 vol of 95% ethanol and the sample was centrifuged at 840*g* (20 °C) for 20 min. The glycogen precipitate was dissolved in 300 μl distilled water and diluted at 1/10. Then, 200 μl of 5% phenol solution and 1 ml of H_2_SO_4_ were added to 200 μl of the glycogen solution obtained, which was then incubated at room temperature for 10 min and at 27 °C for 15 min. Glycogen concentration was determined spectrophotometrically at 490 nm.

### Mitochondrial DNA measurement

Total DNA or RNA was extracted from the gastrocnemius muscle using a QIAamp DNA or RNA mini kit (Qiagen). Genomic (ATP synthase beta) and mitochondrial DNA (NADH dehydrogenase subunit 5) were quantified by quantitative real-time polymerase chain reaction (qPCR; primer sequences are listed in Additional file 1: Table S3). qPCR was performed using KAPA 2× SYBR green master mix on an Miniopticon thermocycler. Cycling conditions were 1 cycle at 98 °C for 30 s following by 40 cycles at 95 °C for 1 s and 60 °C for 15 s. The fusion index was measured for increments of 0.5 °C every 5 s (starting at 65 °C and finishing at 95 °C). Each sample was run in duplicate. Results were expressed using the comparative cycle threshold (Ct) with genomic DNA as the control. The relative changes in the expression level of mitochondrial DNA were calculated by the ΔΔCt formula.

### mRNA analysis

Firstly, 10 ng of each complementary DNA (cDNA) was initially pre-amplified with Taqman PreAmp Master Mix (Applied Biosystems) and a pool containing all the primers (Additional file 1: Table S3) targeting all the genes (200 nM each). To prepare samples for loading into the IFC, a mix was prepared consisting of 440 μl 2X TaqMan Master Mix (Applied 430,976), 44 μl 20× DNA Binding Dye Sample Loading Reagent (Fluidigm 100-3738), 44 μl 20X Evagreen (Biotium 31,000) and 132 μl TE (Tris10mM, EDTA 1mM). Then, 6 μl of this mix was dispensed into each well of a 96-well assay plate. Next, 2 μl of the pre-amplified cDNA sample was added to each well and the plate was briefly vortexed and centrifuged. Following priming of the IFC (Integrated Fluidic Circuit) in the IFC Controller HX, 5 μl of the cDNA sample plus reagent mix were dispensed to each sample inlet of the 96.96 IFC. For the assays, 5 μl of each 10X Assay (5 μM of each primer) were dispensed to each detector inlet of the 96.96 IFC. After loading the assays and samples into the IFC in the IFC Controller HX, the IFC was transferred to the BioMark and PCR was performed using the following thermal protocol: thermal mix at 50 °C for 2 min, 70 °C for 30 min, 25 °C for 10 min, hot start at 50 °C for 2 min, 95 °C for 10 min, 35 cycles of PCR (95 °C for 15 s and 60 °C for 60 s), and melting analysis. The results were analyzed using the Fluidigm real-time PCR analysis software v.4.1.2 and expressed using the comparative cycle threshold (Ct) with tubulin mRNA expression as the control.

### Primary culture of human myoblasts

A quadriceps muscle biopsy from one healthy male adult (30 years old) was taken at the Centre Hospitalier Universitaire Lapeyronie (Montpellier, France). The volunteer signed informed written consent after being described the protocol. Myoblasts were purified from muscle biopsies and were grown on collagen-coated dishes in Dulbecco’s modified Eagle’s medium/F12 with 10% fetal bovine serum (FBS), 0.1% Ultroser G and 1 ng/ml of human basic FGF (Fibroblast Growth Factor) (growth medium) as previously described [67]. For transient siRNA transfections, REDD1 (siREDD1) and negative control (siCTRL) Silencer Select pre-designed siRNAs were purchased from Life Technologies (France). Human myoblasts were transfected with siREDD1 (#S29168, Ambion) and siCTRL as previously described [67] and harvested 48–72 h after transfection. The mean inhibition was 81.4 ± 2.3%. ATP content was quantified by CellTiter-Glo Luminescent Cell Viability/ATP Assay kit (Promega), and normalized by DNA content. For protein synthesis labelling, puromycin (Sigma-Aldrich) was diluted in cell culture media (final concentration 5 μg/ml) for 30 min. Western blots with anti-puromycin antibody (1:2000) were normalized using the Biorad stain-free method.

### Duo-link proximity ligation in situ assay

Cells were fixed 30 min at room temperature with 4% formaldehyde and permeabilized for 10 min at room temperature with phosphate-buffered saline 0.1% Triton X100. PLA was performed according to the Duolink II in situ PLA kit (Sigma-Aldrich) protocol. Nuclei were stained with Hoechst and fluorescence was analyzed with a fluorescence ApoTome microscope (Zeiss). We used the Blobfinder software (Centre for Image Analysis, Uppsala University) to quantify the spot/nucleus signal. For skeletal muscle, in situ PLA was performed on paraffin-embedded sections of gastrocnemius using a bright-field revelation. The nuclei in longitudinal sections were stained and IP3R/VDAC spots were quantified at 20× magnification (ten fields per muscle).

### Statistics

Results are presented as mean ± standard error of mean. All data were tested for normality through skewness and kurtosis or homoscedasticity analyses and then analyzed by Student’s *t*-test or two-way ANOVA (with genotype and stress condition as the two factors). A Fisher's least significant difference post hoc analysis was used to determine differences between groups when ANOVA was significant. Statistical significance was set to *p* < 0.05.

## Additional files

Additional file 1: **Table S1.** Classical markers of hypoxia exposure. **Table S2.** List of antibodies. **Table S3.** Primers used for real-time qPCR. (DOCX 17 kb)

Additional file 2: **Figure S1.** Same as Fig. 1 with all raw data. **Figure S2.** Atrophying program in REDD1 KO muscles after hypoxia exposure. **Figure S3.** REDD1 deletion did not disrupt redox status of skeletal muscle in normoxic or hypoxic mice. **Figure S4.** REDD1 KO mice display an attenuated decrease in Akt/mTOR phosphorylation under energetic stress. **Figure S5.** REDD1 localizes in crude mitochondria after running exercise. **Figure S6.** REDD1 deletion does not alter the respiration capacity of isolated mitochondria. **Figure S7.** REDD1 overexpression does not alter citrate synthase protein expression. **Figure S8.** PRAS40 and mTOR localize in the crude mitochondrial fraction from skeletal muscle. **Figure S9.** Protein synthesis under energetic stress in human myoblasts depleted for REDD1. **Figure S10.** mTOR and HKII activity correlates with basal O_2_ consumption of myoblasts. **Figure S11.** Increase in mitophagy markers following intense running exercise in REDD1 KO mice. (PPTX 11020 kb)

Additional file 3: Individual data values for all experiments in an Excel file. Data sets are sorted by figure. (XLSX 35 kb)
