# Supplementary material for: Glucocorticoid-dependent REDD1 expression reduces muscle metabolism to enable adaptation under energetic stress
Source: BMC Biol. 2018 Jun 12;16:65. doi: 10.1186/s12915-018-0525-4 (PMC5998563; doi:10.1186/s12915-018-0525-4)
Supplement: Supplementary file 1 — Table S1. Classical markers of hypoxia exposure. Table S2. List of antibodies. Table S3. Primers used for real-time qPCR. (DOCX 17 kb) [file 12915_2018_525_MOESM1_ESM.docx]

**Table S1. Classical markers of hypoxia exposure.**

|  | **WT-N** | **WT-H** | **KO-N** | **KO-H** |
| --- | --- | --- | --- | --- |
| Heart weight (mg) | 137.3 ± 4 | 165.6 ± 7.6** | 142.6 ± 3.2 | 166.0 ± 6.4** |
| Hematocrit (%) | 42.0 ± 1.8 | 72.6 ± 1.6*** | 41.6 ± 1.2 | 72.3 ± 1.9*** |
| Food intake (g) | 8.17 ± 0.29 | 5.3 ± 0.5*** | 8.21 ± 0.26 | 5.29 ± 0.32*** |

N and H, normoxic and hypoxic groups, respectively. **p<0.01, ***p<0.001 *vs.* corresponding normoxic group.

**Table S2. List of antibodies.**

| Protein | Application | Provider | | Reference |
| --- | --- | --- | --- | --- |
| REDD1 | WB | ProteinTech Group | | 10638-1-AP, RRID:AB_2245711 |
| α-tubulin | WB | Sigma-Aldrich | | T6199, RRID:AB_477593 |
| Histone-H3 | WB | Cell signaling Technology | 4499, RRID:AB_10544537 | |
| Citrate Synthase | WB | Genetex | | GTX110624, RRID:AB_1950045 |
| IP3R | WB | BD Biosciences | | 610313, RRID:AB_397705 |
| COX-I (mtCO1) | WB | Abcam | | ab14705, RRID:AB_2084810 |
| Myc (Mouse) | PLA | Santa Cruz | | sc-40, RRID:AB_627268 |
| Myc (Rabbit) | PLA | Cell signaling Technology | | 2278, RRID:AB_490778 |
| IP3R | PLA | Santa Cruz | | sc-28614, RRID:AB_2296450 |
| VDAC | PLA/IF | Abcam | | ab14734, RRID:AB_443084 |
| GRP75 | PLA | Santa Cruz | | sc-133137, RRID:AB_2120468 |
| Phospho-Akt T308 | WB | Cell signaling Technology | | 13038, RRID:AB_2629447 |
| Akt | WB | Cell signaling Technology | | 9272, RRID:AB_329827 |
| Phospho-PRAS40 T246 | WB | Cell signaling Technology | | 13175S |
| PRAS40 | WB/IF/IP | Cell signaling Technology | | 2691S, RRID:AB_2225033 |
| UCP3 | WB | Abcam | | ab3477, RRID:AB_2304253 |
| Calreticulin | WB | Cell signaling Technology | | 12238, RRID:AB_2688013 |
| mTOR | WB/IF | Cell signaling Technology | | 2983, RRID:AB_2105622 |
| Hexokinase II | WB | Cell signaling Technology | | 2867, RRID:AB_2232946 |
| Phospho-GSK3αβ S9 | WB | Cell signaling Technology | | 9331, RRID:AB_329830 |
| GSK3αβ | WB | Cell signaling Technology | | 5676, RRID:AB_10547140 |
| Phospho-AMPK T172 | WB | Cell signaling Technology | | 2535, RRID:AB_331250 |
| AMPK | WB | Cell signaling Technology | | 5832, RRID:AB_10624867 |
| Phospho-4EBP1 T37/46 | WB | Cell signaling Technology | | 2855, RRID:AB_560835 |
| 4EBP1 | WB | Cell signaling Technology | | 9644, RRID:AB_2097841 |
| Phospho-rpS6 S240/244 | WB | Cell signaling Technology | | 5364, RRID:AB_10694233 |
| rpS6 | WB | Cell signaling Technology | | 3944, RRID:AB_2146239 |
| Phospho-ULK1 S317 | WB | Cell signaling Technology | | 12753, RRID:AB_2687883 |
| ULK1 | WB | Cell signaling Technology | | 8054, RRID:AB_11178668 |
| BNIP3 | WB | Abcam | | ab10433, RRID:AB_2066656 |

IF: immunofluorescence; IP: immunoprecipitation; PLA: proximity ligation assay; WB: western blot.

**Table S3. Primers used for real-time qPCR.**

| **Gene** | **Forward primer (5’-3’)** | **Reverse primer (5’-3’)** |
| --- | --- | --- |
| PGC-1α | GGACATGTGCAGCCAAGACTCT | CACTTCAATCCACCCAGAAAGCT |
| PGC-1β | GAGGTCAAGCTCTGGCAAGT | GCTCTCGTCCTTCTTCCTCA |
| NRF1 | ggtgggggacagatagtcct | atgctcacagggatctggac |
| NRF2 | ccgctacaccgactacgatt | accttcatcaccaacccaag |
| TFAM | agggagctaccagaagcaga | tgacttggagttagctgctcttt |
| REDD1 | ccagagaagagggccttga | ccatccaggtatgaggagtctt |
| REDD2 | gagcaagatccactttgccta | aggcgggtacaataacagca |
| FOXO1 | gctgggtgtcaggctaagag | agggcatctttggactgct |
| foxo3a | ggaaatgggcaaagcaga | aaacggatcactgtccacttg |
| MAFbx | agtgaggaccggctactgtg | gatcaaacgcttgcgaatc |
| muRF1 | tcctgcagagtgaccaagg | ggcgtagagggtgtcaaac |
| BNIP3 | cctgtcgcagttgggttc | gaagtgcagttctacccaggag |
| lc3 | AGCTTTGAACAAAGAGTGGAAGA | CTCCCCCTTGTATCGCTCTA |
| LAMP2A | GTGACAAAAGGACAGTATTCTACAGC | CCAATAAAATAAGCCAGCAACA |
| GABARAP | GCGAGAAAATCCGAAAGAAA | AGATCAGAAGGCACCAGGTATT |
| cathepsinL | ACAGAAGACTGTATGGCACGAA | GGATCATTCTCATGTTCTTCTCC |
| GPx | ggttcgagcccaattttaca | cccaccaggaacttctcaaa |
| TRX | tgaagctgatcgagagcaag | gaagtccaccacgacaagc |
| TXNIP | atcccagataccccagaagc | tgagagtcgtccacatcgtc |
| ATP synthase β | GGTTCATCCTGCCAGAGACTA | AATCCCTCATCGAACTGGAC |
| ND5 | GGCAGACGAACAAGACATCCGAAA | GCTAGGCGTTTGATTGGGTT |
| α-tubulin | ctggaacccacggtcatc | gtggccacgagcatagttatt |
