# Supplementary material for: Glucocorticoid-dependent REDD1 expression reduces muscle metabolism to enable adaptation under energetic stress
Source: BMC Biol. 2018 Jun 12;16:65. doi: 10.1186/s12915-018-0525-4 (PMC5998563; doi:10.1186/s12915-018-0525-4)
Supplement: Supplementary file 2 — Figure S1. Same as Fig. 1 with all raw data. Figure S2. Atrophying program in REDD1 KO muscles after hypoxia exposure. Figure S3. REDD1 deletion did not disrupt redox status of skeletal muscle in normoxic or hypoxic mice. Figure S4. REDD1 KO mice display an attenuated decrease in Akt/mTOR phosphorylation under energetic stress. Figure S5. REDD1 localizes in crude mitochondria after running exercise. Figure S6. REDD1 deletion does not alter the respiration capacity of isolated mitochondria. Figure S7. REDD1 overexpression does not alter citrate synthase protein expression. Figure S8. PRAS40 and mTOR localize in the crude mitochondrial fraction from skeletal muscle. Figure S9. Protein synthesis under energetic stress in human myoblasts depleted for REDD1. Figure S10. mTOR and HKII activity correlates with basal O2 consumption of myoblasts. Figure S11. Increase in mitophagy markers following intense running exercise in REDD1 KO mice. (PPTX 11020 kb) [file 12915_2018_525_MOESM2_ESM.pptx]

## Slide 1
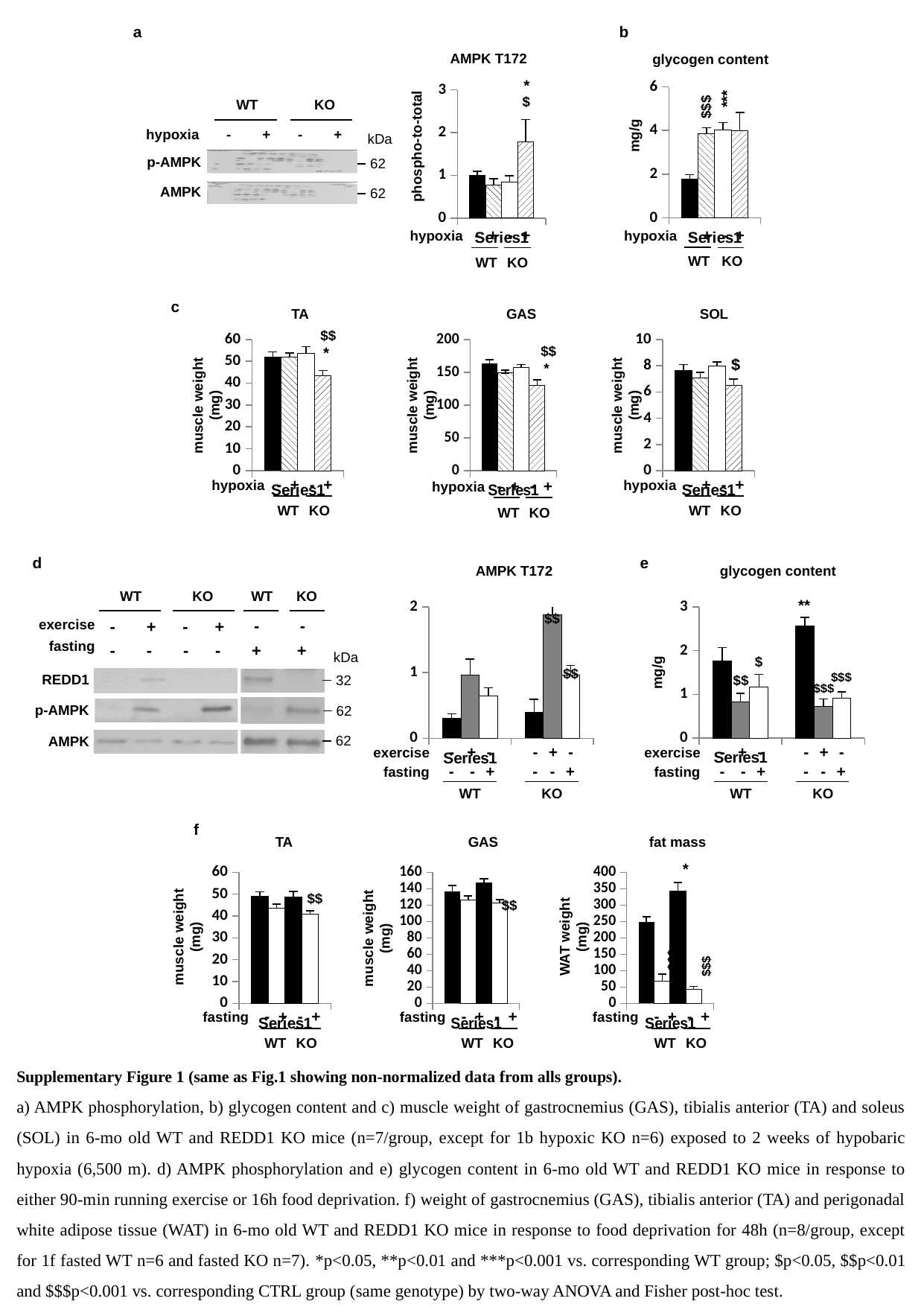

### Chart
| Category | | | | |
|---|---|---|---|---|
| | 1.0 | 0.763 | 0.832 | 1.783 |a
b
### Chart
| Category | | | | |
|---|---|---|---|---|
| | 1.781122070388567 | 3.843050745499765 | 4.0187040843416755 | 4.006296448921206 |AMPK T172
glycogen content
WT
KO
hypoxia - + - +
p-AMPK
AMPK
kDa
62
62
mg/g
phospho-to-total
hypoxia - + - +
hypoxia - + - +
WT KO
WT KO
c
### Chart
| Category | | | | |
|---|---|---|---|---|
| | 52.093 | 52.057 | 53.807 | 43.307 |
### Chart
| Category | | | | |
|---|---|---|---|---|
| | 163.65714285714287 | 149.52857142857144 | 157.02857142857144 | 129.99999999999997 |
### Chart
| Category | | | | |
|---|---|---|---|---|
| | 7.6499999999999995 | 7.078571428571428 | 7.958333333333333 | 6.529999999999999 |TA
GAS
SOL
$$
*
$$
*
$
muscle weight (mg)
muscle weight (mg)
muscle weight (mg)
hypoxia - + - +
hypoxia - + - +
hypoxia - + - +
WT KO
WT KO
WT KO
d
e
AMPK T172
glycogen content
### Chart
| Category | | | |
|---|---|---|---|
| | 1.7668318905594522 | 0.825119050881374 | 1.1665 |
| | 2.5630145708603416 | 0.7279607898379963 | 0.915835950364823 |
### Chart
| Category | | | |
|---|---|---|---|
| | 0.30561742332321495 | 0.9602555701652483 | 0.6421891153016558 |
| | 0.4048438116845176 | 1.8863675943101101 | 0.968796566414307 |WT KO
WT KO
exercise
fasting
 - + - +
 - - - - + +
REDD1
p-AMPK
AMPK
-
-
32
62
62
kDa
**
$$
$
mg/g
$$
$$$
$$
$$$
exercise - + - - + -
 fasting - - + - - +
WT KO
exercise - + - - + -
 fasting - - + - - +
WT KO
f
### Chart
| Category | | | | |
|---|---|---|---|---|
| | 48.95 | 43.583 | 48.758 | 40.764 |
### Chart
| Category | | | | |
|---|---|---|---|---|
| | 136.325 | 126.483 | 147.292 | 122.95 |
### Chart
| Category | | | | |
|---|---|---|---|---|
| | 249.133 | 68.383 | 342.05 | 43.543 |TA
GAS
fat mass
*
$$
$$
muscle weight (mg)
WAT weight (mg)
muscle weight (mg)
$$$
$$$
 fasting - + - +
 fasting - + - +
 fasting - + - +
WT KO
WT KO
WT KO
Supplementary Figure 1 (same as Fig.1 showing non-normalized data from alls groups).
a) AMPK phosphorylation, b) glycogen content and c) muscle weight of gastrocnemius (GAS), tibialis anterior (TA) and soleus (SOL) in 6-mo old WT and REDD1 KO mice (n=7/group, except for 1b hypoxic KO n=6) exposed to 2 weeks of hypobaric hypoxia (6,500 m). d) AMPK phosphorylation and e) glycogen content in 6-mo old WT and REDD1 KO mice in response to either 90-min running exercise or 16h food deprivation. f) weight of gastrocnemius (GAS), tibialis anterior (TA) and perigonadal white adipose tissue (WAT) in 6-mo old WT and REDD1 KO mice in response to food deprivation for 48h (n=8/group, except for 1f fasted WT n=6 and fasted KO n=7). *p<0.05, **p<0.01 and ***p<0.001 vs. corresponding WT group; $p<0.05, $$p<0.01 and $$$p<0.001 vs. corresponding CTRL group (same genotype) by two-way ANOVA and Fisher post-hoc test.

## Slide 2
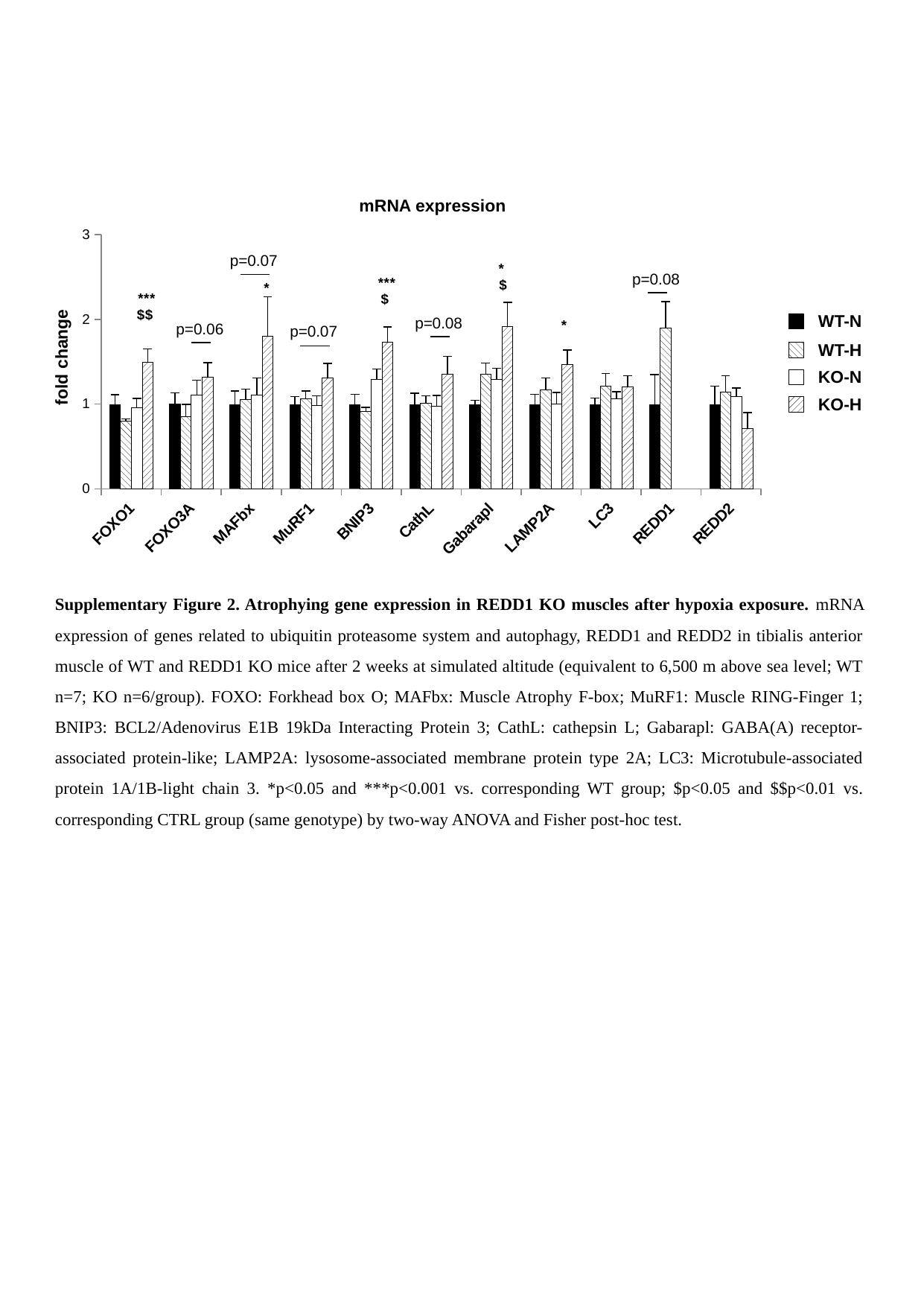

mRNA expression
### Chart
| Category | | | | |
|---|---|---|---|---|
| FOXO1 | 1.0 | 0.797368463097637 | 0.9576112997544736 | 1.494344538684345 |
| FOXO3A | 0.9999999999999999 | 0.8526586133341986 | 1.1017326168871386 | 1.314985330801953 |
| MAFbx | 0.9999999999999998 | 1.0521481356896838 | 1.1056153338172485 | 1.8007 |
| MuRF1 | 1.0 | 1.0631730540753497 | 0.9817424556753629 | 1.3075129890517065 |
| BNIP3 | 1.0000000000000002 | 0.9106359763425403 | 1.2903208924605525 | 1.729683609224416 |
| CathL | 1.0 | 1.013 | 0.97510407571075 | 1.3525066063803879 |
| Gabarapl | 1.0000000000000002 | 1.3509723114667724 | 1.288339275338025 | 1.9121615529394456 |
| LAMP2A | 0.9999999999999999 | 1.1697415539934617 | 1.0048824559628369 | 1.4697580044583696 |
| LC3 | 1.0000000000000002 | 1.2091701584786656 | 1.060961874947879 | 1.203875697906246 |
| REDD1 | 1.0 | 1.9 | None | None |
| REDD2 | 1.0000000000000002 | 1.1424939229878999 | 1.0907821566440354 | 0.7103606410072971 |p=0.07
p=0.08
WT-N
p=0.08
p=0.06
p=0.07
WT-H
fold change
KO-N
KO-H
Supplementary Figure 2. Atrophying gene expression in REDD1 KO muscles after hypoxia exposure. mRNA expression of genes related to ubiquitin proteasome system and autophagy, REDD1 and REDD2 in tibialis anterior muscle of WT and REDD1 KO mice after 2 weeks at simulated altitude (equivalent to 6,500 m above sea level; WT n=7; KO n=6/group). FOXO: Forkhead box O; MAFbx: Muscle Atrophy F-box; MuRF1: Muscle RING-Finger 1; BNIP3: BCL2/Adenovirus E1B 19kDa Interacting Protein 3; CathL: cathepsin L; Gabarapl: GABA(A) receptor-associated protein-like; LAMP2A: lysosome-associated membrane protein type 2A; LC3: Microtubule-associated protein 1A/1B-light chain 3. *p<0.05 and ***p<0.001 vs. corresponding WT group; $p<0.05 and $$p<0.01 vs. corresponding CTRL group (same genotype) by two-way ANOVA and Fisher post-hoc test.

## Slide 3
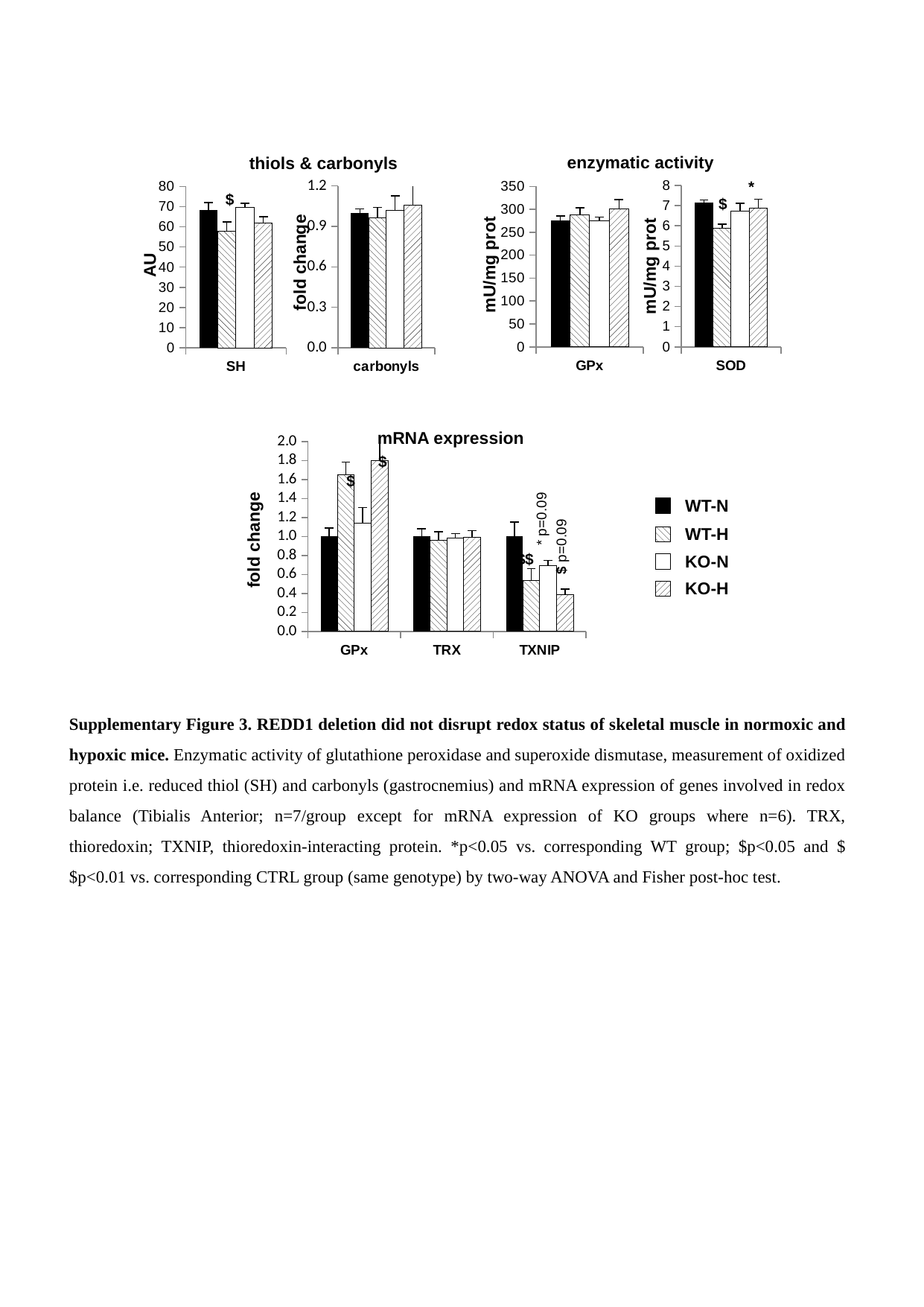

enzymatic activity
thiols & carbonyls
### Chart
| Category | | | | |
|---|---|---|---|---|
| SOD | 7.146844112125382 | 5.86173098293645 | 6.729811427654373 | 6.89135335806403 |
### Chart
| Category | | | | |
|---|---|---|---|---|
| GPx | 276.5437834048009 | 287.4901379307603 | 274.64924202839853 | 301.229171710971 |
### Chart
| Category | | | | |
|---|---|---|---|---|
| SH | 68.14283350570514 | 57.78526692576505 | 69.64953533541083 | 61.928416379677294 |
### Chart
| Category | | | | |
|---|---|---|---|---|
| carbonyls | 1.0 | 0.9612483892137625 | 1.0195089870413498 | 1.0544178334412642 |fold change
mU/mg prot
AU
mU/mg prot
mRNA expression
### Chart
| Category | | | | |
|---|---|---|---|---|
| GPx | 1.0000000000000002 | 1.6521898024838755 | 1.1364881482683593 | 1.8021723104847116 |
| TRX | 1.0 | 0.96 | 0.98 | 0.99 |
| TXNIP | 1.0 | 0.5382121308761564 | 0.6953805149628487 | 0.38798121252473644 |WT-N
WT-H
fold change
KO-N
KO-H
Supplementary Figure 3. REDD1 deletion did not disrupt redox status of skeletal muscle in normoxic and hypoxic mice. Enzymatic activity of glutathione peroxidase and superoxide dismutase, measurement of oxidized protein i.e. reduced thiol (SH) and carbonyls (gastrocnemius) and mRNA expression of genes involved in redox balance (Tibialis Anterior; n=7/group except for mRNA expression of KO groups where n=6). TRX, thioredoxin; TXNIP, thioredoxin-interacting protein. *p<0.05 vs. corresponding WT group; $p<0.05 and $$p<0.01 vs. corresponding CTRL group (same genotype) by two-way ANOVA and Fisher post-hoc test.

## Slide 4
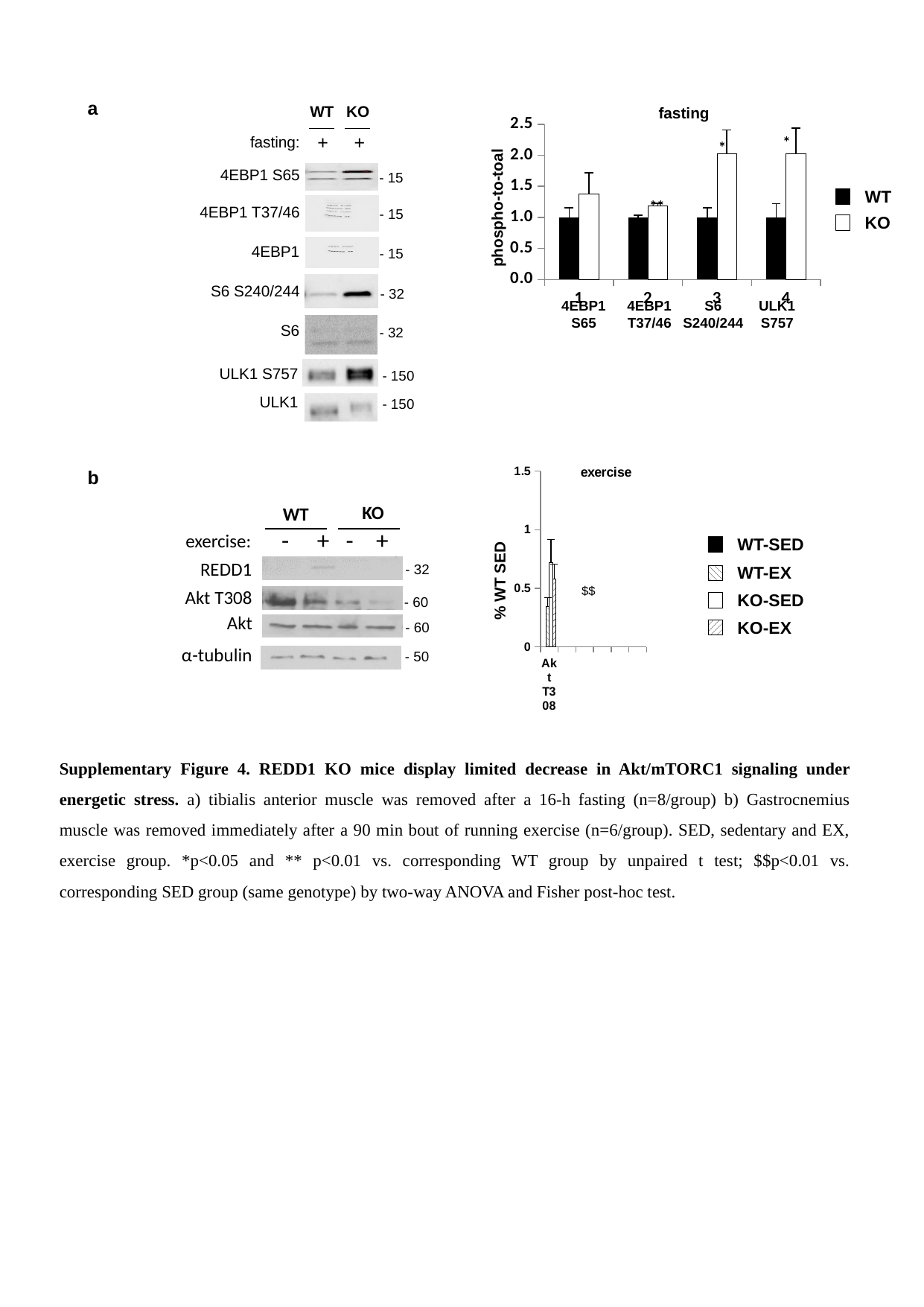

a
WT
KO
fasting
*
phospho-to-toal
**
### Chart
| Category | | |
|---|---|---|4EBP1
S65
4EBP1
T37/46
S6
S240/244
ULK1
S757
*
WT
KO
 + +
fasting:
4EBP1 S65
- 15
4EBP1 T37/46
- 15
4EBP1
- 15
S6 S240/244
- 32
S6
- 32
ULK1 S757
- 150
ULK1
- 150
### Chart
| Category | | | | |
|---|---|---|---|---|
| Akt T308 | 0.9999999999999999 | 0.34689549727705254 | 0.7192398250668622 | 0.5785691302654837 |b
KO
WT
 - + - +
exercise:
REDD1
- 32
Akt T308
- 60
Akt
- 60
α-tubulin
- 50
WT-SED
WT-EX
% WT SED
$$
KO-SED
KO-EX
Supplementary Figure 4. REDD1 KO mice display limited decrease in Akt/mTORC1 signaling under energetic stress. a) tibialis anterior muscle was removed after a 16-h fasting (n=8/group) b) Gastrocnemius muscle was removed immediately after a 90 min bout of running exercise (n=6/group). SED, sedentary and EX, exercise group. *p<0.05 and ** p<0.01 vs. corresponding WT group by unpaired t test; $$p<0.01 vs. corresponding SED group (same genotype) by two-way ANOVA and Fisher post-hoc test.

## Slide 5
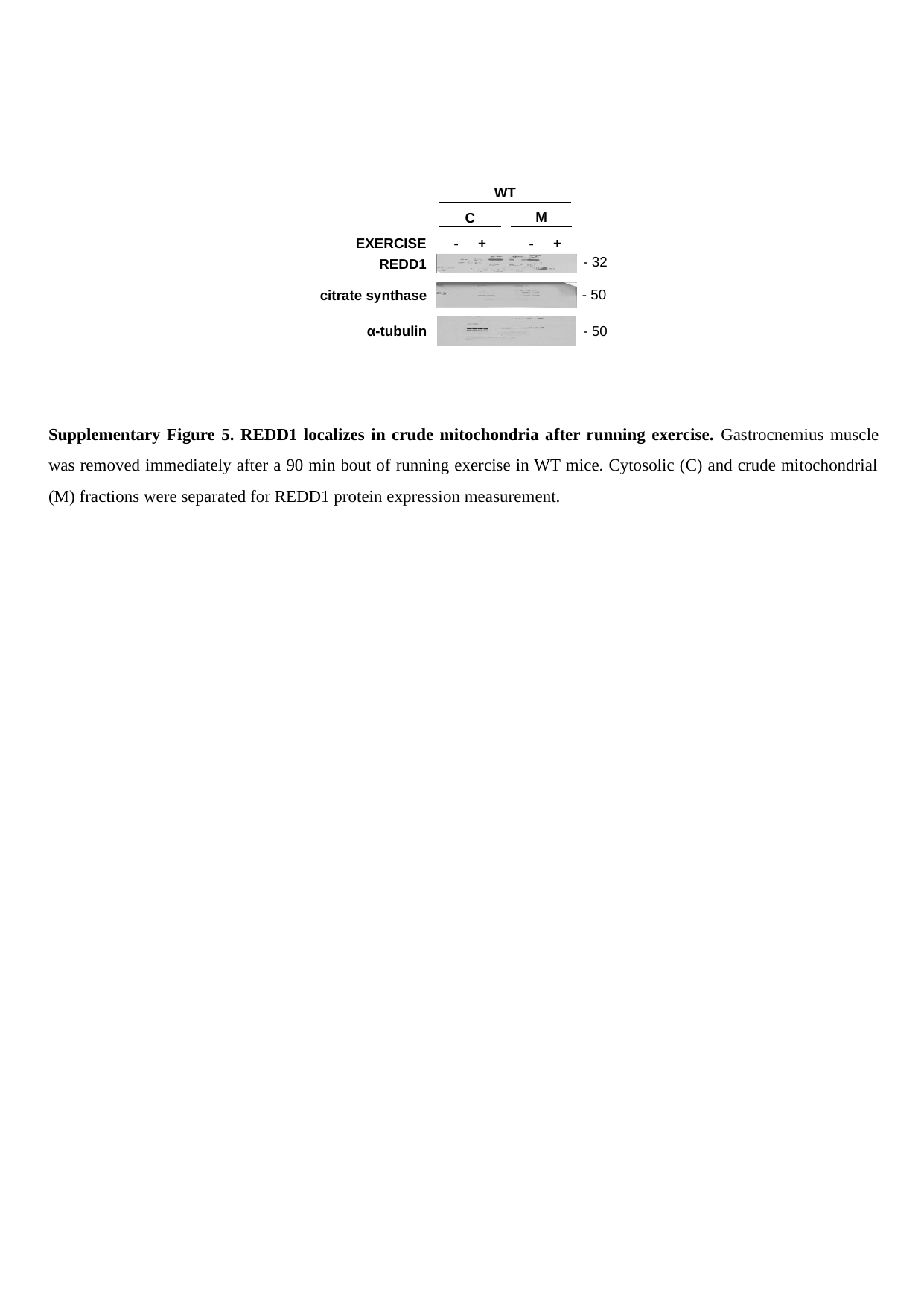

WT
M
C
EXERCISE
 - + - +
REDD1
- 32
- 50
citrate synthase
α-tubulin
- 50
Supplementary Figure 5. REDD1 localizes in crude mitochondria after running exercise. Gastrocnemius muscle was removed immediately after a 90 min bout of running exercise in WT mice. Cytosolic (C) and crude mitochondrial (M) fractions were separated for REDD1 protein expression measurement.

## Slide 6
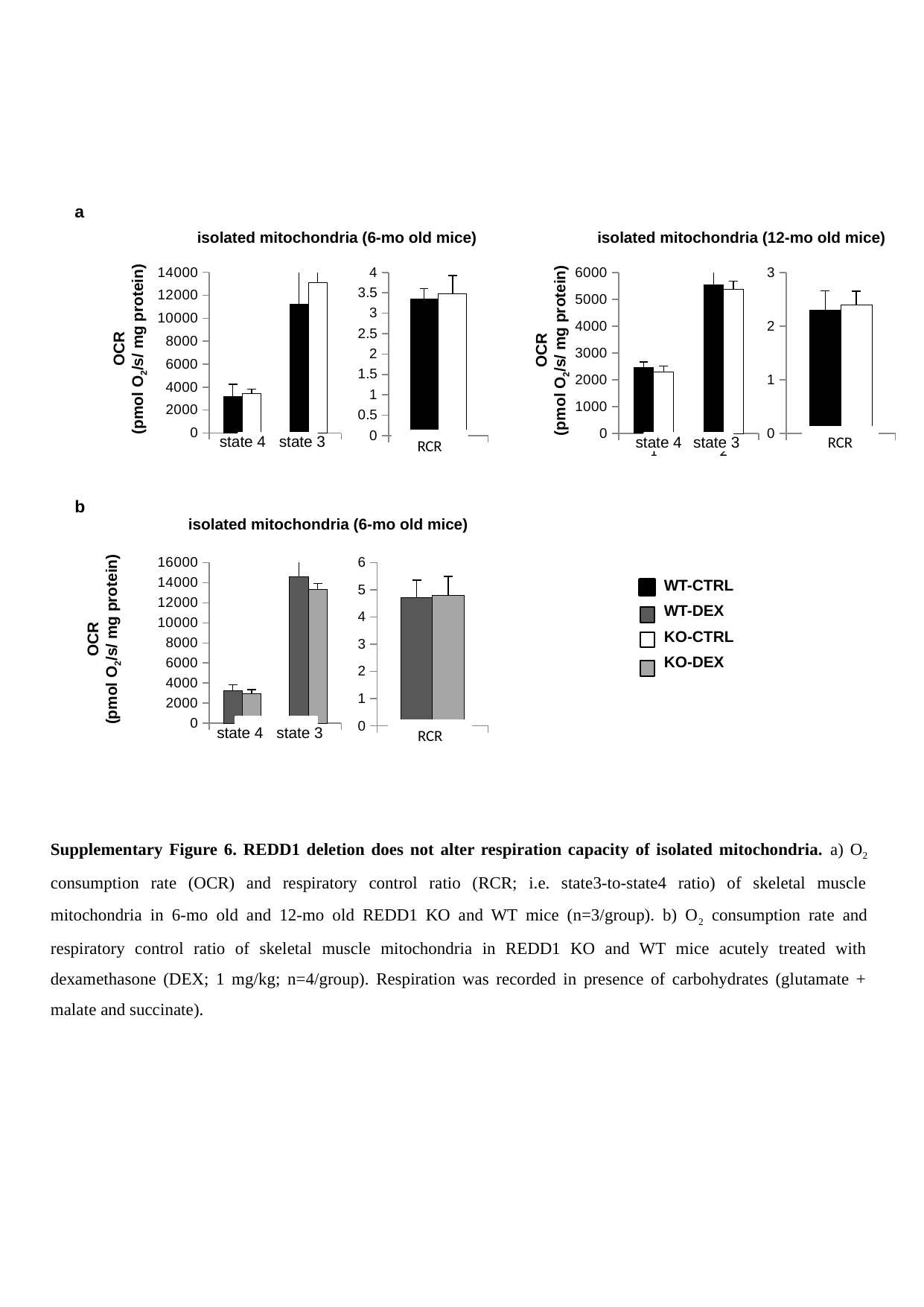

a
isolated mitochondria (6-mo old mice)
isolated mitochondria (12-mo old mice)
### Chart
| Category | wt | ko |
|---|---|---|
### Chart
| Category | | |
|---|---|---|
### Chart
| Category | wt | ko |
|---|---|---|
### Chart
| Category | | |
|---|---|---|OCR
(pmol O2/s/ mg protein)
OCR
(pmol O2/s/ mg protein)
state 4
state 3
state 4
state 3
RCR
RCR
b
isolated mitochondria (6-mo old mice)
### Chart
| Category | wt | ko |
|---|---|---|
### Chart
| Category | | |
|---|---|---|WT-CTRL
WT-DEX
KO-CTRL
KO-DEX
OCR
(pmol O2/s/ mg protein)
state 4
state 3
RCR
Supplementary Figure 6. REDD1 deletion does not alter respiration capacity of isolated mitochondria. a) O2 consumption rate (OCR) and respiratory control ratio (RCR; i.e. state3-to-state4 ratio) of skeletal muscle mitochondria in 6-mo old and 12-mo old REDD1 KO and WT mice (n=3/group). b) O2 consumption rate and respiratory control ratio of skeletal muscle mitochondria in REDD1 KO and WT mice acutely treated with dexamethasone (DEX; 1 mg/kg; n=4/group). Respiration was recorded in presence of carbohydrates (glutamate + malate and succinate).

## Slide 7
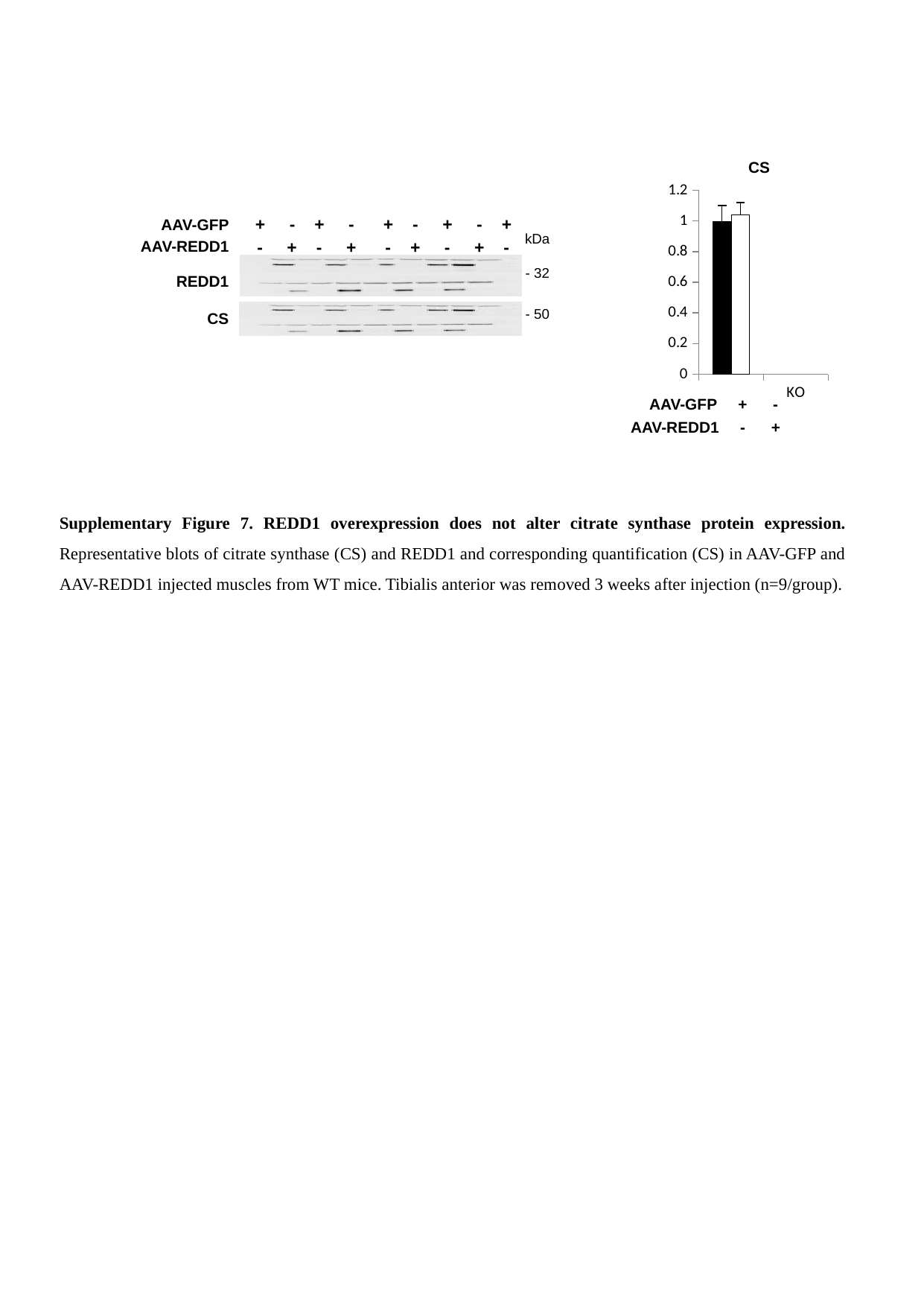

CS
[unsupported chart]
+ - + - + - + - +
AAV-GFP
kDa
AAV-REDD1
- + - + - + - + -
- 32
REDD1
- 50
CS
AAV-GFP + -
AAV-REDD1 - +
Supplementary Figure 7. REDD1 overexpression does not alter citrate synthase protein expression. Representative blots of citrate synthase (CS) and REDD1 and corresponding quantification (CS) in AAV-GFP and AAV-REDD1 injected muscles from WT mice. Tibialis anterior was removed 3 weeks after injection (n=9/group).

## Slide 8
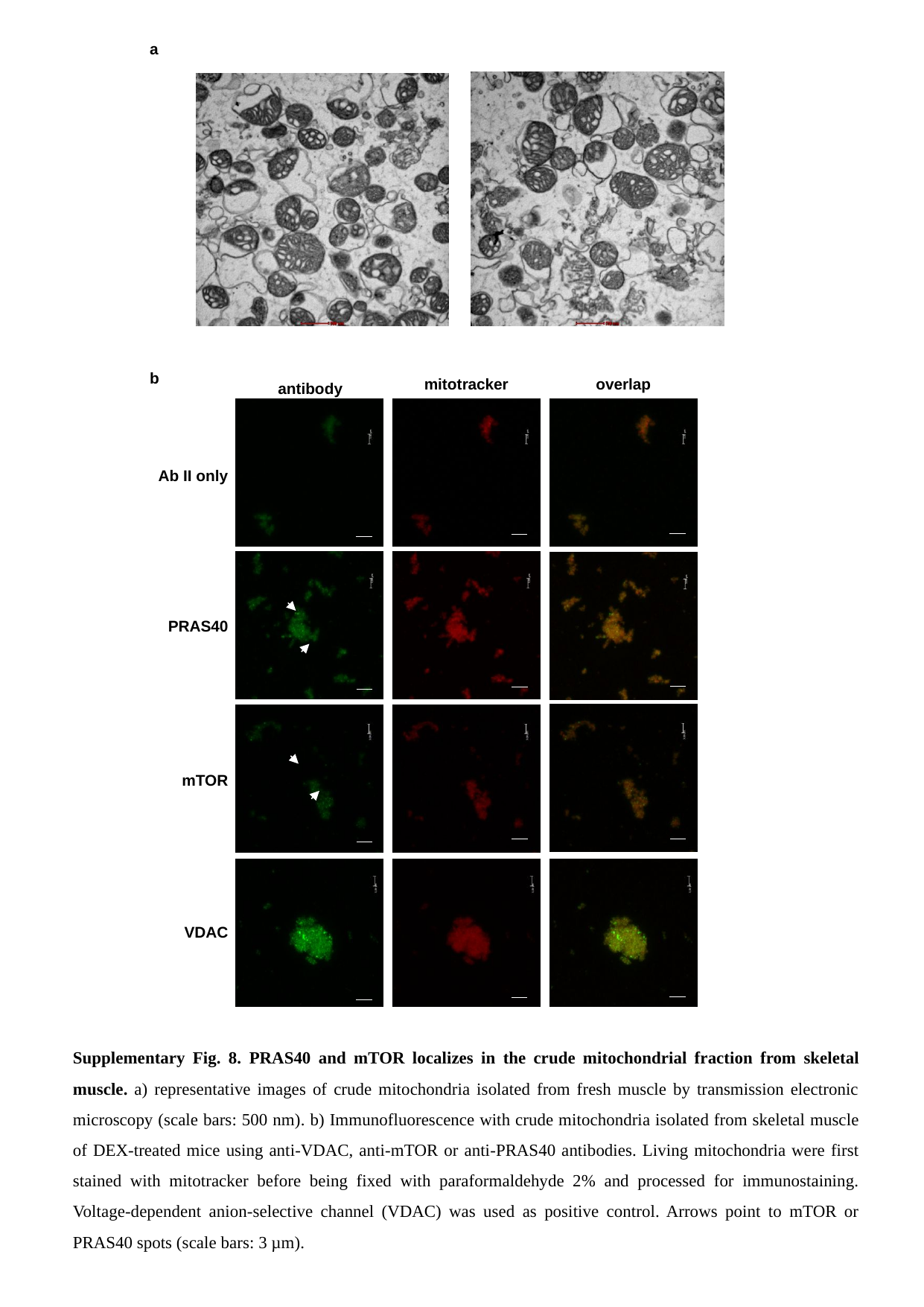

a
b
overlap
mitotracker
antibody
Ab II only
PRAS40
mTOR
VDAC
Supplementary Fig. 8. PRAS40 and mTOR localizes in the crude mitochondrial fraction from skeletal muscle. a) representative images of crude mitochondria isolated from fresh muscle by transmission electronic microscopy (scale bars: 500 nm). b) Immunofluorescence with crude mitochondria isolated from skeletal muscle of DEX-treated mice using anti-VDAC, anti-mTOR or anti-PRAS40 antibodies. Living mitochondria were first stained with mitotracker before being fixed with paraformaldehyde 2% and processed for immunostaining. Voltage-dependent anion-selective channel (VDAC) was used as positive control. Arrows point to mTOR or PRAS40 spots (scale bars: 3 µm).

## Slide 9
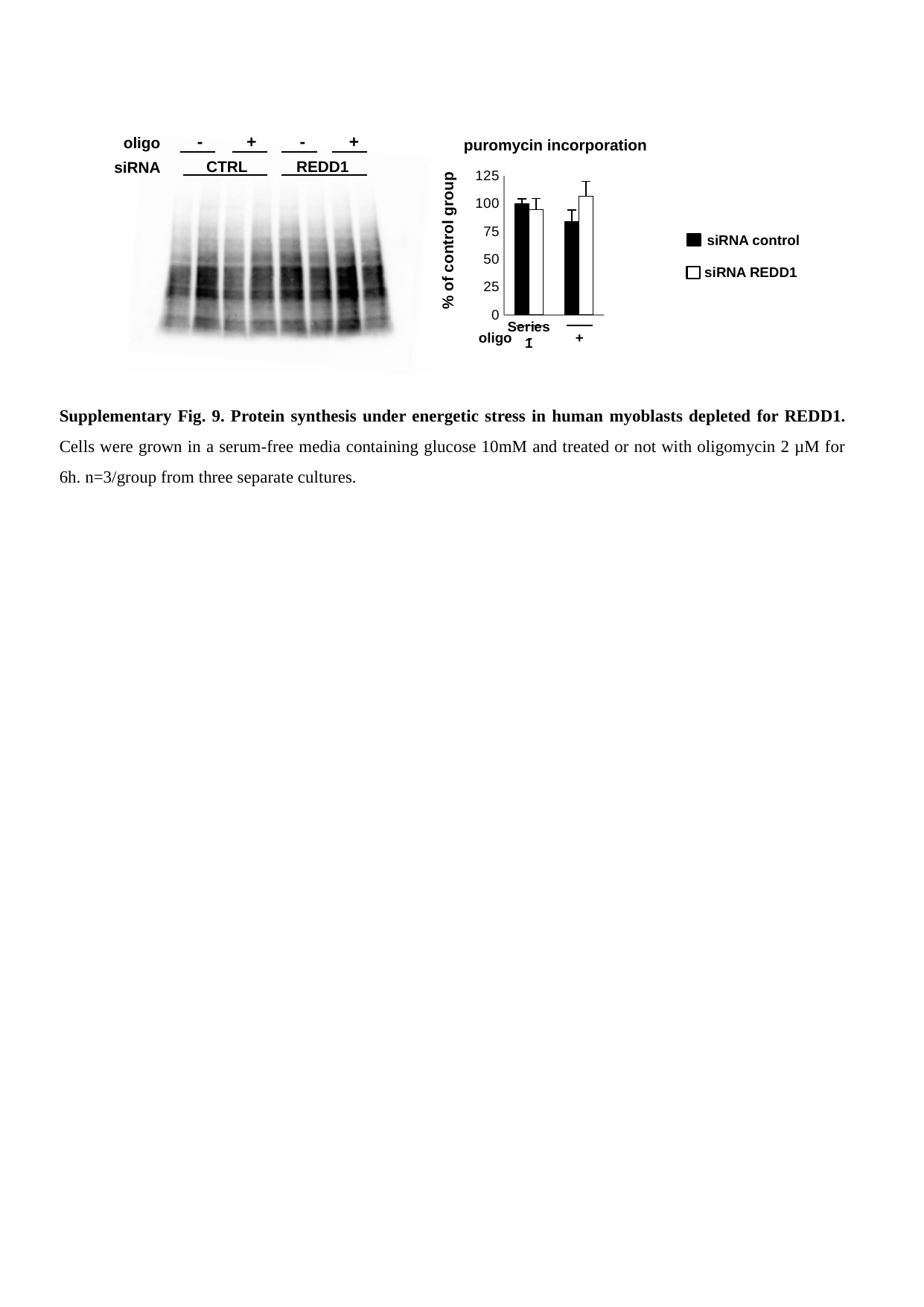

- + - +
oligo
puromycin incorporation
CTRL
REDD1
siRNA
### Chart
| Category | | |
|---|---|---|
| | 100.0 | 94.37834300414976 |
| | 84.08435614162907 | 106.78979029710901 |% of control group
siRNA control
siRNA REDD1
oligo - +
Supplementary Fig. 9. Protein synthesis under energetic stress in human myoblasts depleted for REDD1. Cells were grown in a serum-free media containing glucose 10mM and treated or not with oligomycin 2 µM for 6h. n=3/group from three separate cultures.

## Slide 10
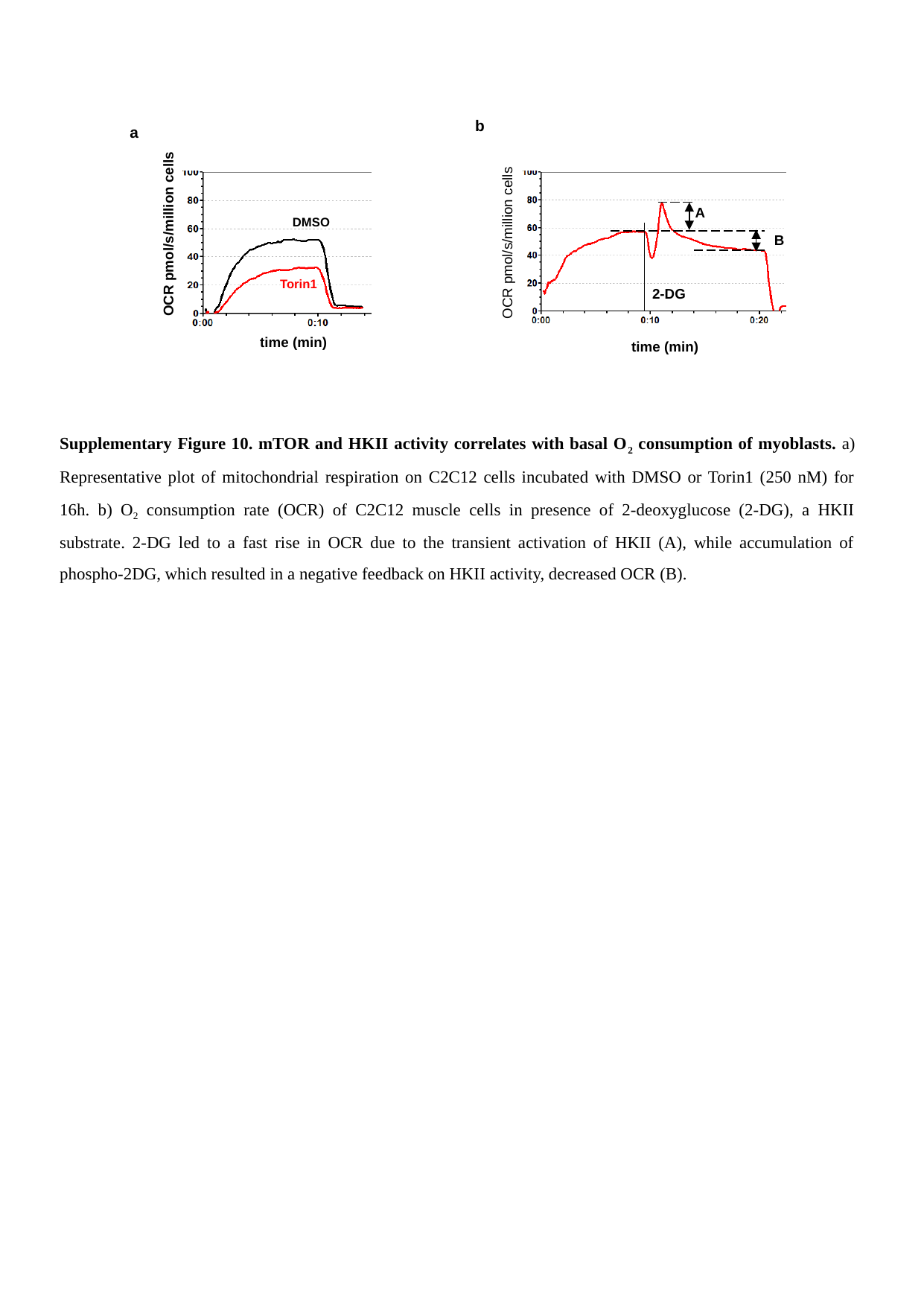

b
a
A
DMSO
OCR pmol/s/million cells
B
OCR pmol/s/million cells
Torin1
2-DG
time (min)
time (min)
Supplementary Figure 10. mTOR and HKII activity correlates with basal O2 consumption of myoblasts. a) Representative plot of mitochondrial respiration on C2C12 cells incubated with DMSO or Torin1 (250 nM) for 16h. b) O2 consumption rate (OCR) of C2C12 muscle cells in presence of 2-deoxyglucose (2-DG), a HKII substrate. 2-DG led to a fast rise in OCR due to the transient activation of HKII (A), while accumulation of phospho-2DG, which resulted in a negative feedback on HKII activity, decreased OCR (B).

## Slide 11
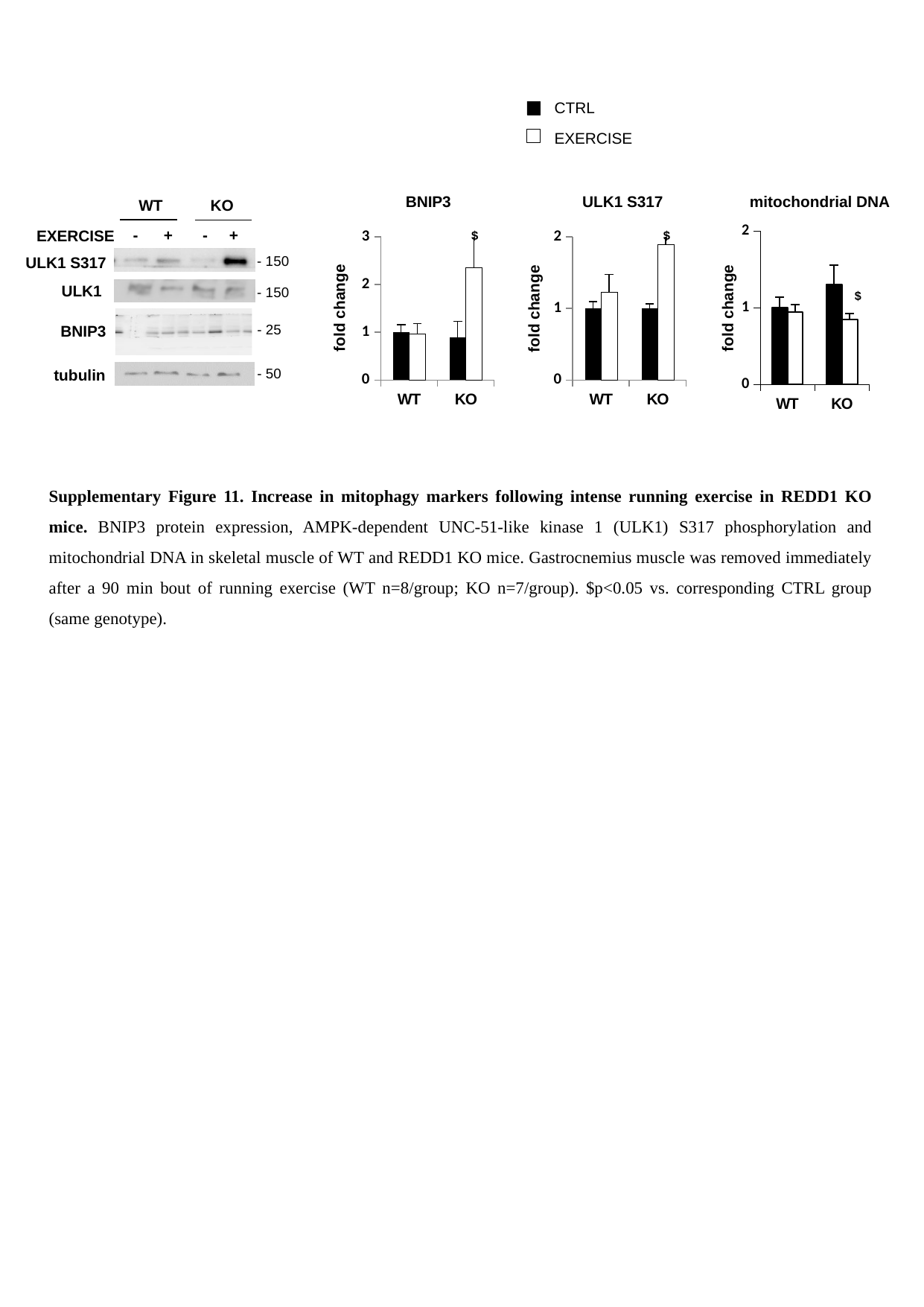

CTRL
EXERCISE
ULK1 S317
BNIP3
mitochondrial DNA
WT
KO
 - + - +
EXERCISE
ULK1 S317
ULK1
tubulin
BNIP3
### Chart
| Category | SED | EX |
|---|---|---|
| WT | 1.0 | 0.94 |
| KO | 1.3 | 0.85 |
### Chart
| Category | | |
|---|---|---|
| WT | 1.0 | 0.959 |
| KO | 0.888 | 2.345 |
### Chart
| Category | | |
|---|---|---|
| WT | 1.0 | 1.222 |
| KO | 1.003 | 1.888 |- 150
- 150
fold change
fold change
fold change
- 25
- 50
Supplementary Figure 11. Increase in mitophagy markers following intense running exercise in REDD1 KO mice. BNIP3 protein expression, AMPK-dependent UNC-51-like kinase 1 (ULK1) S317 phosphorylation and mitochondrial DNA in skeletal muscle of WT and REDD1 KO mice. Gastrocnemius muscle was removed immediately after a 90 min bout of running exercise (WT n=8/group; KO n=7/group). $p<0.05 vs. corresponding CTRL group (same genotype).
